# Functional analysis of a UDP-glucosyltransferase gene contributing to biosynthesis of the flavonol triglycoside in tea plants

**DOI:** 10.1093/hr/uhaf149

**Published:** 2025-05-06

**Authors:** Wen-Wen Zhang, Feng-Yi Xiao, Cun-Yu Li, Hong-Zhiyuan Yang, Dong Zhao, Jian-Hui Ye, Xin-Qiang Zheng, Yue-Rong Liang, Zhou-Tao Fang, Jian-Liang Lu

**Affiliations:** Tea Research Institute, Zhejiang University, Hangzhou 310058, China; Tea Research Institute, Zhejiang University, Hangzhou 310058, China; Tea Research Institute, Zhejiang University, Hangzhou 310058, China; Tea Research Institute, Zhejiang University, Hangzhou 310058, China; Tea Research Institute, Zhejiang University, Hangzhou 310058, China; Tea Research Institute, Zhejiang University, Hangzhou 310058, China; Tea Research Institute, Zhejiang University, Hangzhou 310058, China; Tea Research Institute, Zhejiang University, Hangzhou 310058, China; Tea Research Institute, Zhejiang University, Hangzhou 310058, China; Crop Science Institute, Zhejiang University, Hangzhou 310058, China; Tea Research Institute, Zhejiang University, Hangzhou 310058, China

## Abstract

Flavonol glycosides have many prominent benefits to human health and significant contributions to the growth and development of tea plant as well as the color and taste of tea infusion. In this study, a gene isolated from tea plant was found to encode a 52.2-kDa protein located on the plasma membrane and in the cytoplasm with activity of flavonol glycosyltransferase (CsFGT). The prokaryotically expressed recombinant CsFGT (rCsFGT) exhibited its main glucosyl transfer activity towards rutin to produce quercetin 3-*O*-β-d-glucopyranosyl-(1→3)-α-l-rhamnopyranosyl-(1→6)-β-d-glucopyranoside (Q-g-r-g), and showed a minor galactosyl transfer activity towards delphinidin to produce delphinidin 3-*O* galactoside. The maximum activity of rCsFGT was observed at 30°C and pH 8.0. The main function of rCsFGT seems to be catalysis of the biosynthesis of Q-g-r-g rather than delphinidin 3-*O* galactoside since its affinity and catalytic efficiency are much higher towards rutin than towards delphinidin. Molecular docking and site-directed mutation reveal that amino acid residues G290, E292, R319, and Q352 play important roles in the catalytic specificity of CsFGT. The Q-g-r-g content in leaves of different tea cultivars was significantly correlated with the *CsFGT* expression level. Injection of antisense oligodeoxyribonucleotides remarkably downregulated endogenous *CsFGT* expression and consequently reduced the Q-g-r-g content significantly. These findings will help elucidate the differential accumulation mechanism of flavonol glycosides in different tea germplasms.

## Introduction

Flavonols and flavonol glycosides are key metabolites in *Camellia sinensis* (L.) O. Kuntze, accounting for ~3%–4% of dry weight in the tender leaves. It has been reported that flavonol glycosides make important contributions to the growth and development of tea plants and to resistance against adversities [1]. Meanwhile, these compounds, in particular the flavonol diglycosides (such as rutin) and triglycosides (such as quercetin 3-*O*-β-d-glucopyranosyl-(1→3)-α-l-rhamnopyranosyl-(1→6)-β-d-glucopyranoside), not only can provide distinctively velvet astringency even at a very low concentration [2], but also have many prominent health benefits like antioxidant, antimutagenic, antibacterial, and cardiovascular protective properties [3]. Flavonols in tea plants can mainly be classified into kaempferol, quercetin, and myricetin according to the number of hydroxyl groups on the B ring of the molecule, and are usually glycosylated at 3-OH on the C ring and/or 7-OH on the A ring, but mainly at 3-OH [4]. The flavonol glycosides can be divided into myricetin glycosides, quercetin glycosides, and kaempferol glycosides according to the aglycones, and also into flavonol mono-, di-, and triglycoside(s) based on the number of glycosyl groups. The formation of flavonol glycosides in organisms is catalysed by a group of glycosyltransferases (GTs).

GTs catalyse the formation of glycosidic bonds from a wide range of substrates, and can use monosaccharides, oligosaccharides, proteins, lipids, DNAs, and small organic molecules as acceptors. Family 1 of the GTs, also referred to as uridine diphosphate (UDP)-dependent glycosyltransferases (UGTs), can catalyse glycosylation towards small organic molecules such as secondary metabolites and xenobiotics. The UGTs can use UDP-glucose (UDP-glc), UDP-galactose (UDP-gal), UDP-rhamnose (UDP-rha), UDP-glucuronic acid, UDP-xylose and UDP-mannose as the activated sugar donors [5], and use a wide range of small molecules as sugar acceptors, including anthocyanins, terpenoids, benzoates, phenylpropanoids, saponins, hormones, flavonols, and flavonol glycosides [6]. Plant UGTs have a highly conserved motif of 44 amino acids at the C-terminus responsible for binding the glycosyl groups, named the plant secondary product glycosyltransferase (PSPG) box [4]. Since glycosylation of the metabolites will alter the biological activity, enhance the water solubility, and improve the chemical stability of the compounds [7], considerable research has focused on glycosylation of the target metabolites by recombinant UGTs. Increasing amounts of released plant genomic data provide a massive amount of information about the putative UGTs in model and non-model plants, including ornamentals, economic crops and medical plants [8–11], and many of the UGTs have been cloned and functionally characterized *in vitro*.

Genes involved in the biosynthesis of phenylpropanoids, catechins, and flavonols have been well studied in tea plants; however, few genes associated with the glycosylation of the flavonols have been fully characterized since a large variety of *UGT*s are involved. According to the genomic and transcriptomic analyses, more than 170 *UGT*s might be included in tea plant. To date, only seven *UGT*s contributing to flavonol glycosylation have been isolated and characterized, comprising *CsUGT73A17*, *CsUGT78A15*, *CsUGT78A14*, *CsUGT72AM1*, *CsUGT73A20*, *CsUGT75L12*, and *CsUGT73AC15*. Based on *in vitro* expression tests, UGTs encoded by these genes exhibit a relatively broad substrate spectrum for the flavonol aglycones, and two of them can even use different UDP-sugars as donors. In particular, CsUGT73A20, CsUGT72AM1, CsUGT78A14, and CsUGT78A15 can transfer the activated glucose moiety mainly onto the 3-OH of kaempferol, quercetin, and myricetin; among them, CsUGT73A20 can also contribute to 7-*O*-glucosylation of quercetin and myricetin [5], and CsUGT78A14 and CsUGT78A15 can also use UDP-galactose as sugar donor besides UDP-glucose. CsUGT73A17 can only contribute to the 3-*O*-glycosylation of quercetin [12]. Interestingly, CsUGT75L12 can catalyse trace amounts of kaempferol 3-*O-*glucoside (K-g) or quercetin 3-*O*-glucoside (Q-g) to their 3-*O*, 7-*O*-diglycosides besides mainly contributing to the 7-*O*-glycosylation of kaempferol [13]. Catalysis of CsUGT73AC15 displays some promiscuity and regioselectivity of the substrate, enabling glycosylation of quercetin 3-*O*-rutinoside (Q-g-r or rutin) at multiple sites and kaempferol 3-*O*-rutinoside (K-g-r) at the 7-OH position [14]. In tea plants, flavonols can be converted into their corresponding flavonol mono-, di-, and triglycosides by primary, secondary, and tertiary transglycosylation reactions, and the abundance of the flavonol di- and triglycosides is usually higher than that of the flavonol monoglycosides [15]. Unfortunately, functionally defined genes associated with the biosynthesis of flavonol glycosides are quite limited considering the diversity of these compounds as well as the large number of *UGT*s present in the tea plant genome. In peach, PpUGT91AK6 exhibits significant rhamnosyl transfer activity towards flavonol monoglycosides (K-g and Q-g) to produce the flavonol diglycosides, i.e. K-g-r and Q-g-r [16]. GmFg1 of the soybean can exert glucosyl transfer activity and convert K-g into kaempferol 3-*O*-glucosyl-(1→6)-glucoside, while GmFg2 can catalyse the biosynthesis of K-g-r from K-g and UDP-rha [17]. In addition, quercetin diglycosides can be formed through catalysis of FaUGT71A33 and FaUGT71A34 in *Fragaria × ananassa* [18], while PIUGT3 can govern isoflavonoid 4-*O*, 7-*O*-di-glycosylations in *Pueraria lobata* [19]. Interestingly, the CaUGT3 of *Catharanthus roseus* possesses a continuous glucosyl chain elongation activity and mediates the biosynthesis of flavonol diglycoside, triglycoside, and tetraglycoside in a sequential manner using Q-g and UDP-glc as the initial substrates [20]. However, the genes contributing to the catalysis of the biosynthesis of the flavonol 3-*O* di- and triglycosides in tea plant remain unclear.

Reports showed that the number of hydroxyl groups on the B-ring of catechins and the length of the sugar chain on the C-ring of flavonol glycosides could be used to distinguish the *assamica* and *sinensis* varieties of tea plant; in particular, the *assamica* variety usually could synthesize high levels of epicatechin gallate (ECG) and quercetin 3-*O*-diglycosides (Q-dg) rather than epigallocatechin gallate (EGCG) and quercetin 3-*O*-triglycosides (Q-tg), while the *sinensis* variety could produce high levels of EGCG and Q-tg rather than ECG and Q-dg. Thus, a high ECG/EGCG ratio and a low Q-tg/Q-dg ratio were observed in the *assamica* variety, but a reverse pattern was seen in the *sinensis* variety [15, 21]. In this study, a *flavonol glycosyltransferase* (*CsFGT*) was shown to encode the enzyme catalysing the biosynthesis of quercetin 3-*O*-β-d-glucopyranosyl-(1 → 3)-α-l-rhamnopyranosyl-(1→6)-β-d-glucopyranoside (Q-g-r-g) from Q-g-r in tea plant based on our previous gene screening according to weighted gene co-expression network analysis (WGCNA) between the transcription level of the genes and the content of flavonol glycosides in the different representative cultivars belonging to the *assamica* and *sinensis* varieties. The function of CsFGT was confirmed by experiments *in vitro* and *in vivo*. The catalytic specificity of CsFGT was also explained through molecular docking and confirmed by site-directed mutation.

## Results

### Bioinformatic attributes of CsFGT

Five candidate genes possibly contributing to biosynthesis of the flavonol triglycosides were screened out based on WGCNA analysis between the transcription level of the differentially expressed genes (DEGs) and content of flavonol glycosides in leaves at various position on shoots harvested from ‘Fudingdabaicha’ (FD) and ‘Yunkang 10#’ (YK), which were representative cultivars of the *sinensis* and *assamica* varieties, respectively (Supplementary Data Fig. S1, Supplementary Data Table S1), and these genes were cloned from tea cultivar FD through RT–PCR based on the published ‘Longjing 43’ genomic data [22] using the designed special primers (Supplementary Data Table S2), but only one gene encoded a protein that could catalyse the biosynthesis of the flavonol triglycoside and was named *CsFGT*. The ORF of *CsFGT* consisted of 1410 bp and could be deduced to be a protein with 469 amino acid residues that shared high similarity with homologs in other tea cultivars (‘Longjing43’, ‘Suchazao’, ‘Tieguanyin’, and YK) and wild tea germplasm ‘DASZ’ (Supplementary Data Fig. S2). The deduced protein had a theoretical molecular mass of 52.2 kDa, an isoelectric point of 6.70, and a hydrophobicity of −0.064. The protein included 36.03% α-helixes, 7.25% β-sheets, 42.43% random coils, and 14.29% extended strands in its secondary structure. It was predicted that CsFGT belonged to UGT91Q1 of the glycosyltransferase superfamily according to the UGT nomenclature committee.

**Figure 1:**
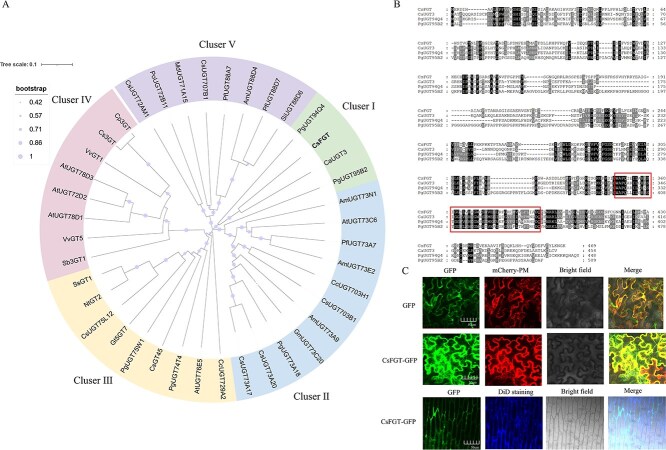
Comparison of CsFGT with other plant UGTs and subcellular localization of CsFGT. (A) Phylogenetic tree of plant UGTs .The tree was constructed using MEGA11 and iTOL with the neighbor-joining method and classified into five clusters (I, II, III, IV, V). CsFGT was in cluster I and is indicated in bold font. GenBank accession numbers of the *UGT*s are given in Supplementary Data Table S3. (B) Amino acid sequence alignment of CsFGT with its homolog in Cluster I. The UGTs in multiple alignment were CaUGT3 (BAH80312.1), PgUGT94Q4 (QEA68984.1), and PgUGT95B2 (AZB52139.1). Alignment was performed using Clustal Omega online analysis software. The red rectangle indicates the PSPG box. (C) Subcellular localization of CsFGT in tobacco leaf cells and onion inner epidermal cells. Scale bar = 50 μm. mCherry-PM was the cell membrane localization marker in tobacco leaf cells, and DiD was the cell membrane fluorescent stain for onion inner epidermal cells.

After comparing CsFGT with several UGTs that had been already identified from other plant species (Supplementary Data Table S3), a phylogenetic tree was constructed using MEGA11 software and the iTOL online tool (https://itol.embl.de/). As shown in Fig. 1A, the UGTs could be divided into five clusters, and CsFGT was included in cluster I with PgUGT94Q4, CaUGT3, and PgUGT95B2. UGTs in cluster I shared 19.40%–23.67% similarity with each other, and possessed the conserved PSPG box in their C-terminus (Fig. 1B). Reports showed that CaUGT3 exhibited continuous glucosyl transfer activity to form the final product quercetin 3-*O*-tetraglycoside in *Catharanthus roseus* [20], PgUGT94Q4 contributed to the biosynthesis of K-g in *Panax ginseng* [23], and PgUGT95B2 had high glycosyl transfer activity towards several flavones and flavonols [24]. The UGTs in cluster II, from diverse species, including *Crocosmia crocosmiiflora*, *Perilla frutescens*, and *Antirrhinum majus*, were all able to catalyse glycosylation of flavonols. Most of the UGTs in cluster IV exhibited activity towards anthocyanins. The UGTs in cluster III and cluster V had a wide range of substrates, including saponins, chalcones, and isoflavones. It seemed that the UGTs in cluster I exhibit significant heterogeneity in their substrates, implying that many more substrates need to be investigated in order to elucidate the characteristics of CsFGT.

*CsFGT* (without stop codon) was fused with *GFP* at its 3′ end and expressed in tobacco leaves and onion inner epidermis in order to reveal its subcellular location. The results showed that the green fluorescent signal was concentrated on the cell membrane and dispersed in the cytoplasm, indicating CsFGT was mainly located on the cell membrane and in the cytoplasm (Fig. 1C).

### Substrate specificity of CsFGT

*CsFGT* was inserted into the expression cassette of pCold-TF vector and expressed in Origami (DE3) through inducement of isopropyl-β-d-thiogalactopyranoside (IPTG) at 16°C (Fig. S3 A). The recombinant protein was separated and purified by interaction of its fused His-tag with the Ni^+^ affinity column. SDS–PAGE showed that the molecular weight of rCsFGT was around 105 kDa, including the His tag (0.8 kDa), trigger factor (48 kDa), trigger factor cut-off polypeptide (4 kDa), and the target CsFGT (52.2 kDa) (Supplementary Data Fig. S3B), which was consistent with the prediction. rCsFGT was verified after being digested with HRV 3C protease (Supplementary Data Fig. S3C and D).

A series of enzymatic reactions was conducted and monitored by HPLC, in which rCsFGT, various sugar donors (UDP-glc, UDP-rh, and UDP-gal) and different sugar acceptors, including flavonols or flavonol glycosides (kaempferol, quercetin, K-g, Q-g, K-g-r, Q-g-r), anthocyanins or anthocyanin glycosides (delphinidin, cyanidin-3-*O*-galactoside, cyanidin-3,5,-*O*-diglucoside, pelargonidin, malvidin), and catechins (EC, EGCG, ECG, GC, EGC), were used (Supplementary Data Fig. S4). rCsFGT exhibited its main glucosyl transfer activity only when Q-g-r and UDP-glc were included in the reaction solution (Supplementary Data Fig. S4F01), and showed minor galactosyl transfer activity towards delphinidin from UDP-gal (Supplementary Data Fig. S4A01).

**Figure 2:**
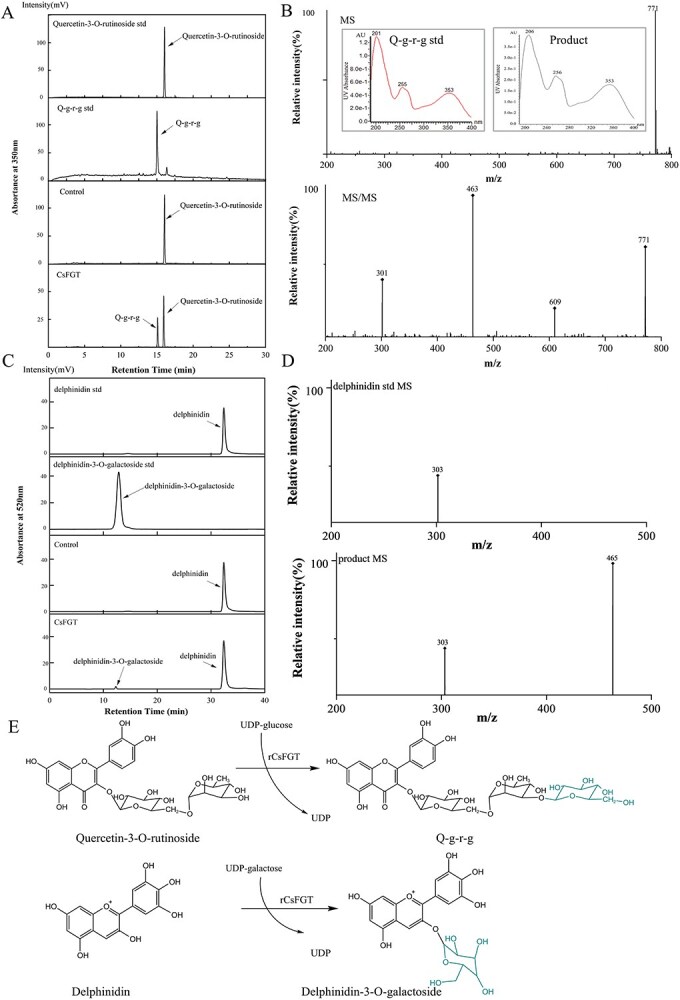
Catalytic characterization of the prokaryotically expressed rCsFGT. (A) HPLC spectrum of the compound references and reaction products catalysed by rCsFGT and control protein at 350 nm. (B) UPLC–DAD–MS/MS analysis of the reaction product catalysed by rCsFGT (above: UV absorbance and MS total scan at ES^−^ mode; below: MS daughter scan of the parent ion *m*/*z* 771). (C) HPLC spectrum of the compound references and reaction products catalysed by rCsFGT and control protein at 520 nm. (D) UPLC–DAD–MS/MS analysis of the reaction product catalysed by rCsFGT (above: MS total scan signal of delphinidin in ES^+^ mode; below: MS daughter scan signal of product using the parent ion *m*/*z* 465). (E) Catalytic reaction of rCsFGT: biosynthesis of Q-g-r-g through glucosylation of Q-g-r and biosynthesis of delphinidin 3-*O* galactoside through galactosylation of delphinidin.

Glucosyl transfer activity of CsFGT towards Q-g-r was comprehensively investigated. Catalytic properties of rCsFGT with or without fusion of the trigger factor were compared, but no significant difference was observed, suggesting that fusion of the trigger factor would not influence the substrate specificity and catalytic efficiency, and HRV3C protease digestion was not necessary for enzymatic research (Supplementary Data Fig. S4F01). The retention time of the product was shorter than that of the substrate Q-g-r but the same as that of Q-g-r-g in the HPLC spectrum (Fig. 2A). The product possessed three UV absorption peaks at 353, 256, and 206 nm, and had a parent ion at *m*/*z* 771 [M–H]^−^ with daughter ions at *m*/*z* 609 [M–(glc moiety–H_2_O)–H]^−^, 463 [M–(glc moiety–H_2_O)–(rha moiety–H_2_O) –H]^−^ and 301 [M–(glc moiety–H_2_O)–(rha moiety–H_2_O)–(glc moiety–H_2_O)–H]^−^ (Fig. 2B), implying the product was a quercetin triglycoside. After comparison with the chromatographic behavior and DAD–MS/MS information on the reference compound, the product was identified as Q-g-r-g (Fig. 2B). The quercetin triglycoside was then purified according to our previous method [15] and confirmed as Q-g-r-g by ^1^H-NMR and ^13^C-NMR measurements. The NMR signals of Q-g-r-g are shown in Supplementary Data Table S4. Obviously, this CsFGT of tea plant can catalyse the biosynthesis of Q-g-r-g from Q-g-r and UDP-glc.

Similarly, galactosyl transfer activity of CsFGT towards delphinidin was also studied. The chromatographic behavior of the product was the same as that of the reference compound delphinidin 3-*O* galactoside (Fig. 2C). It had a parent ion at *m*/*z* 465 [M]^+^ with daughter ions at *m*/*z* 303 [M–(gal moiety–H_2_O)]^+^, which was same as the delphinidin signal (Fig. 2D), revealing that the product was delphinidin 3-*O* galactoside. Thus, CsFGT could also catalyse the biosynthesis of delphinidin 3-*O* galactoside from delphinidin and UDP-gal, besides the catalysis of Q-g-r-g biosynthesis (Fig. 2E).

### Catalytic conditions and enzymatic kinetics of rCsFGT

**Figure 3:**
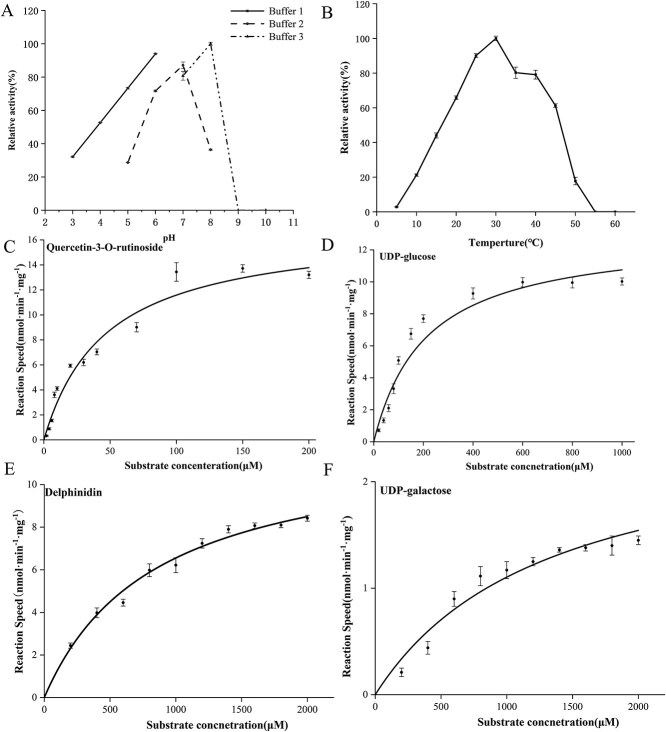
Effects of acidity, temperature, and substrate concentration on the catalytic properties of rCsFGT. (A) Effect of acidity on enzymatic activity of rCsFGT. Reactions were performed for 60 min at 30°C and various pH values (buffer 1, citric acid/ citrate buffer; buffer 2, Tris–HCl buffer; buffer 3, phosphate buffer). (B) Effect of temperature on enzymatic activity of rCsFGT. Reactions were performed for 60 min at pH 8.0 and different temperatures. (C) Effect of Q-g-r concentration on reaction speed of rCsFGT. Substrates were 1 mM UDP-glucose and various Q-g-r concentrations. (D) Effect of UDP-glc concentration on reaction speed of rCsFGT. Substrates were 100 μM Q-g-r and different UDP-glc concentrations, and reactions were conducted at pH 8.0 and 30°C for 10 min. (E) Effect of delphinidin cocentration on reaction speed of rCsFGT. Substrates were 1 mM UDP-galactose and various delphinidin concentrations. (F) Effect of UDP-galactose concentration on reaction speed of rCsFGT. Kinetic parameters were determined after fitting the experimental data to the Michaelis–Menten equation through Origin software.

Using Q-g-r and UDP-glc as substrates, reaction efficiency was tested at different pH values (3–10) and various temperatures (5–60°C). Results showed that the catalysis efficiency of rCsFGT was dramatically influenced by the buffer type and acidity. In particular, along with an increase in pH value, catalysis efficiency of the rCsFGT increased linearly in citrate buffer (pH 3–6), and showed an increasing followed by a decreasing tendency in Tris–HCl buffer (pH 5–8) and phosphate buffer (pH 7–10). Peak values in Tris–HCl and phosphate buffer were at pH 7.0 and 8.0, respectively. Comparatively, the highest glycosyl transfer activity of rCsFGT could be achieved at pH 8.0 (Fig. 3A). The tests also revealed that the best catalysis efficiency was observed at 30°C (Fig. 3B).

The kinetic tests were performed at pH 8.0 (50 mM phosphate buffer) and 30°C for 10 min, and 10 μg purified rCsFGT and various concentrations of substrates were used (Fig. 3C and D). After monitoring the concentrations of the substrates and product, kinetic parameters were obtained by fitting the test data to the Michaelis equation. The results showed that the *K*_m_ values of rCsFGT were 50.3 and 209.7 μM for Q-g-r and UDP-glc, respectively, and the *K*_cat_/*K*_m_ values were 6.1 × 10^5^ and 1.1 × 10^5^ s^−1^ M^−1^ for these two substrates (Table 1). It was clear that CsFGT could efficiently catalyse glucosyl transfer from UDP-glc to Q-g-r. Similarly, the enzyme kinetic parameters of rCsFGT towards delphinidin and UDP-gal were examined (Fig. 3E and F); comparatively higher *K*_m_ but lower *K*_cat_/*K*_m_ values were observed in catalysis of CsFGT towards delphinidin and UDP-gal (Table 1). Clearly, the affinity and catalytic efficiency of rCsFGT towards rutin and UDP-glc were much greater than towards delphinidin and UDP-gal.

**Table 1:** Kinetic parameters of prokaryotically expressed rCsFGT.

Substrate	*K*_m_ (μM)	*V*_max_(nmol min^−1^ mg^−1^)	*K*_cat_ (s^−1^)	*K*_cat/_ *K*_m_ (s^−1^ M^−1^)
Q-g-r	50.3 ± 9.7	17.6 ± 1.8	30.8	6.1 × 10^5^
UDP-glc	209.7 ± 43.9	13.0 ± 1.1	22.8	1.1 × 10^5^
Del	902.5 ± 109.0	12.4 ± 0.6	21.7	2.4 × 10^4^
UDP-gal	1143.3 ± 313.5	2.4 ± 0.3	4.2	3.1 × 10^3^

Q-g-r, quercetin-3-*O*-rutinoside; UDP-glc, UDP-glucose; Del, delphinidin; UDP-gal, UDP-galactose. Reactions were performed at pH 8.0 and 30°C.

### Catalysis active sites of CsFGT

**Figure 4:**
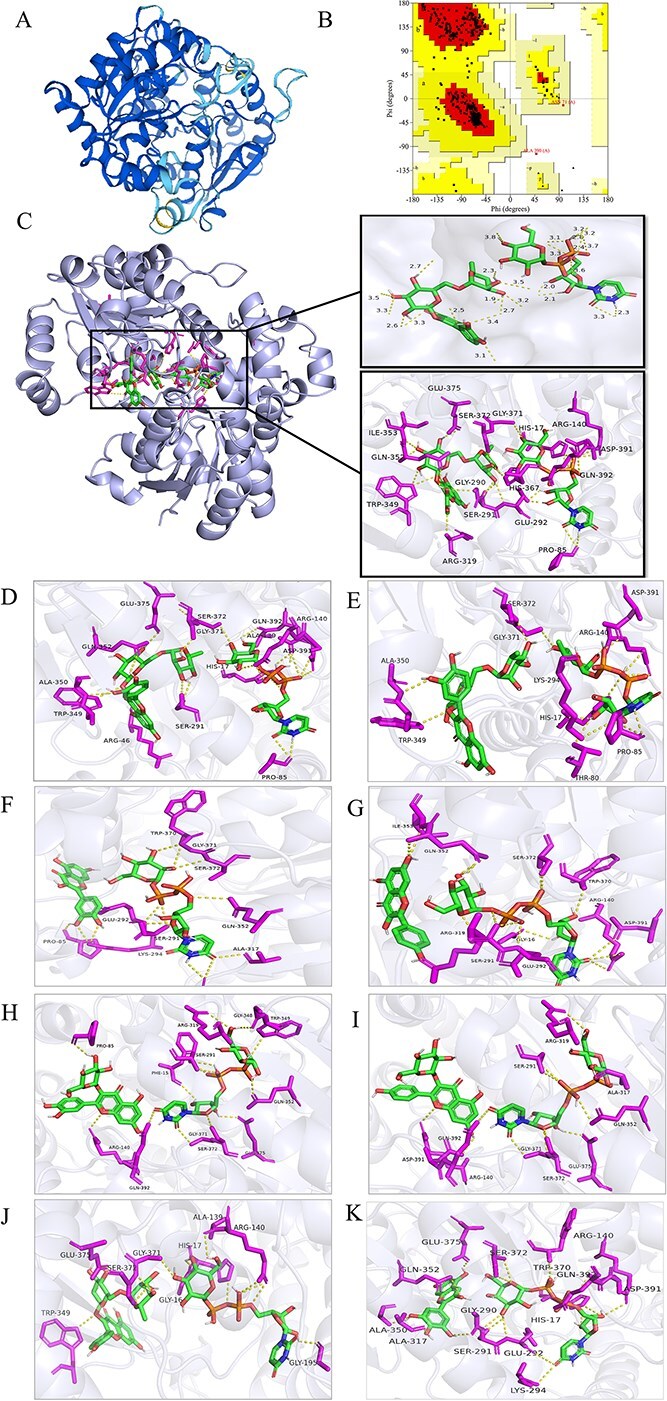
Results of homology modeling and molecular docking for CsFGT. (A) 3D model of CsFGT. (B) Ramachandran plot of CsFGT. (C–K) Molecular docking of CsFGT with different substrates: (C) with Q-g-r and UDP-glc; (D) with Q-g-r and UDP-gal; (E) with Q-g-r and UDP-rha; (F) with quercetin and UDP-glc; (G) with kaempferol and UDP-glc; (H) with Q-g and UDP-glc; (I) with K-g and UDP-glc; (J) with K-g-r and UDP-glc; (K) with delphinidin and UDP-gal.

Homology modeling was performed to elucidate the spatial structure of CsFGT, and global model quality estimation (GMQE) of CsFGT ranged from 0 to 1 and qualitative model energy analysis (QMEAN) changed from −4 to 0, indicating the established model was successful (Fig. 4A). According to calculation of φ and ψ after Ramachandran mapping, the amino acids in the optimal permissible region of the model were as high as 91.3%, suggesting the predicted structure of CsFGT was highly reliable and conformed to the rules of stereochemistry (Fig. 4B).

Molecular docking was carried out to highlight the interaction sites between the enzyme and sugar donor (UDP-glc)/acceptor (Q-g-r) pair. According to the docking results, UDP-glc was predicted to be bound by seven active amino acids, i.e. the uracil moiety by P85 and E292; the diphosphate moiety by the H17, R140, D391, and Q392; and the glucosyl moiety by G371. Q-g-r (rutin) was predicted to be bound by 11 amino acids, i.e. the B ring of the quercetin moiety by G290, R319, and Q352; the C ring of the quercetin moiety by W349; and the rutinosyl moiety by S291, E292, W349, Q352, I353, H367, S372, and E375 (Fig. 4C). During the catalytic process of CsFGT, active pocket formation might depend on the interaction of some key single amino acid residues with different domains of the same substrate (such as W349, Q352) and with two substrates simultaneously (such as E292), as well as on the synergistic binding of the continuous amino acid residues with substrates (such as G290-S291-E292, G371-S372, and D391-Q392). Docking was also performed using other sugar donor/acceptor pairs, including UDP-gal/Q-g-r, UDP-rha/Q-g-r, UDP-glc/quercetin, UDP-glc/kaempferol, UDP-glc/Q-g, UDP-glc/K-g, and UDP-glc/K-g-r (Fig. 4D–J). Relatively loose reaction pockets were observed because of lack of common interaction amino acid sites for the two substrates, leading to a mismatched spatial orientation and/or greater reaction difficulty between these sugar donor/acceptor pairs. When UDP-gal and delphinidin were docked with CsFGT, the sugar donor and acceptor moiety was drawn close by E375, S372, Q352, A350, and A317, as well as successive amino acid residues, G290, S291, and E292 (Fig. 4K). Furthermore, comparisons revealed that G290, S291, E292, R319, and Q352 residues might be the key interaction sites for formation of the compact pocket and exhibition of substrate selectivity.

**Figure 5:**
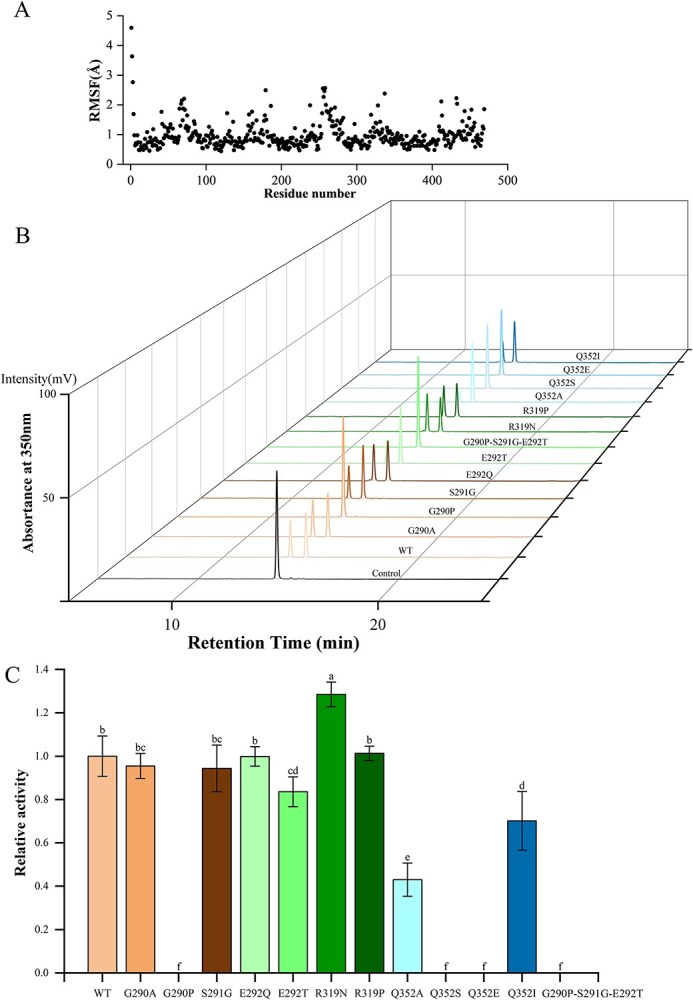
Molecular dynamics of wild-type CsFGT and catalytic activity of rCsFGT mutants towards Q-g-r and UDP-glc. (A) RMSF values of wild-type CsFGT. (B) Detection of reaction product catalysed by different rCsFGT mutants by HPLC. (C) Relative enzymatic activity of different mutants in comparison with wild-type rCSFGT.

FoldX was used to check key amino acid residues for potential function by comparing the free energy difference between wild-type protein and virtually mutated ones. When G290 and E292 were replaced with other random amino acids, the free energies of the mutated proteins all increased obviously and the protein structures became more unstable. Meanwhile, the structure of mutated proteins also become unstable when R319 was replaced with other amino acids except proline. However, after mutation of S291 into other amino acids, the variants possessed lower free energies and became more stable, while mutation of Q352 resulted in increased or decreased free energies accordingly (Table 2). Calculation of root mean square fluctuation (RMSF) revealed that the RMSF values of residues G290, E292, R319, and Q352 were all very small, around 0.5 Å (Fig. 5A), indicating that these amino acid residues were inflexible and had small degrees of freedom due to tight interaction with the substrates during the simulation process, which was consistent with the previous molecular docking and FoldX results. Therefore, it might be true that the amino acid residues G290, E292, R319, and Q352 were very important for the catalytic characteristics of CsFGT.

**Table 2:** Change in free energy of virtually mutated CsFGT.

Residue	Free energy change (Kcal mol^−1^)^a^				
substitution	G290	S291	E292	R319	Q352
A	2.85	−0.18	1.31	0.99	0.25
C	3.84	−0.28	0.98	1.27	0.56
D	4.46	−0.27	0.79	1.41	−0.40
E	3.92	−0.95		1.41	0.03
F	2.89	−0.72	0.41	0.53	−0.15
G		0.389	1.30	1.31	1.05
I	5.94	−1.31	0.68	0.86	−0.98
K	3.39	−1.11	1.07	0.69	0.22
L	3.46	−1.24	0.25	0.65	−0.59
M	3.50	−1.48	0.33	0.44	−1.03
N	4.76	−1.10	1.09	1.43	0.55
P	5.15	−0.47	1.48	−0.44	0.04
Q	4.09	−1.02	0.07	1.12	
R	3.39	−1.03	0.93		−0.46
S	2.26		1.29	1.37	0.81
T	5.04	−0.08	1.44	1.30	0.25
V	6.15	−0.61	1.20	1.35	−0.28
W	3.82	−0.39	0.45	0.25	0.78
Y	2.30	−0.63	0.51	0.55	0.11

a Free energy change (ΔΔG) was calculated according to the equation ΔΔG = ΔG_variant_ − ΔG_wild-type_

**Figure 6:**
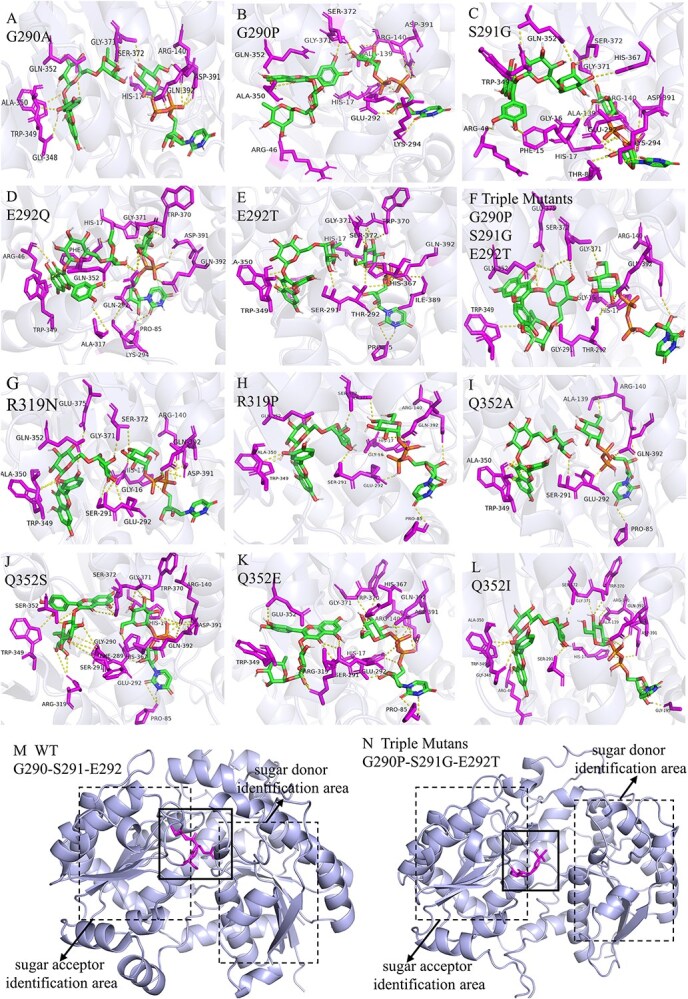
Results of molecular docking between mutated proteins and substrates. (A) Mutant G290A. (B) Mutant G290P. (C) Mutant S291G. (D) Mutant E292Q. (E) Mutant E292T. (F) Mutant G290P–S291G–E292T. (G) Mutant R319N. (H) Mutant R319P. (I) Mutant Q352A. (J) Mutant Q352S. (K) Mutant Q352E. (L) Mutant Q352I. (M) Modeling of wild-type CsFGT (G290–S291–E292) and its loop region structure. (N) Modeling of mutant G290P–S291G–E292T and its loop region structure. The box with solid line represents the loop region where the three successive amino acid residues (G290, S291, E292) were located.

To verify the role of these newly identified key residues, site-directed mutagenesis of CsFGT was conducted (Supplementary Data Fig. S5). The results showed that some mutants, including G290P, Q353S, Q352E, and G290P-S291T-E292T, completely lost catalytic activity and E292T, Q352A, and Q352I mutants exhibited significantly decreased activity; however, the mutant R319N had significantly enhanced activity and the other mutants showed similar activity in comparison with the wild type (Fig. 5B and C). Interestingly, the docking results showed that the mutants with activity unchanged or enhanced (G290A, S291G, E292Q, R319N, and R319P) could provide two amino acid residues to interact with the two hydroxyl groups on the B ring of rutin, but mutants with activity lost and significantly decreased (G290P, E292T, Q352A, Q352S, Q352E, and Q352I) could only provide one amino acid residue to interact with the rutin B ring (Fig. 6A–L), indicating that interaction between the two amino acid residues of CsFGT and the two free hydroxyl groups on the B ring of rutin was one of the most important prerequisites for forming the active reaction pocket and favorable spatial orientation. It is worth mentioning that, according to our results, complete or partial loss of catalytic activity caused by mutation of Q352 was closely related to the weakened interactions of the mutant with the sugar acceptor rutin instead of with the sugar donor, although Q352 was included in the PSPG box, which had been generally considered to be responsible for binding the sugar donor. Meanwhile, the triple mutation G290P-S291G-E292T resulted in a loose loop region, leading to a spatial obstacle in glycosyl group transfer and handover between sugar acceptor and donor (Fig. 6M and N). In other words, the three consecutive amino acids (G290, S291, E292) played an important role in forming the compact pocket in the loop domain beneficial in joining the sugar acceptor and donor regions together [25]. Thus, the successive amino acid residues G290, S291, E292 in the loop domain and Q352 in the PSPG box of CsFGT were particularly important for specific interaction with rutin to generate the Q-g-r-g.

### Relationship between *CsFGT* expression and Q-g-r-g content

**Figure 7:**
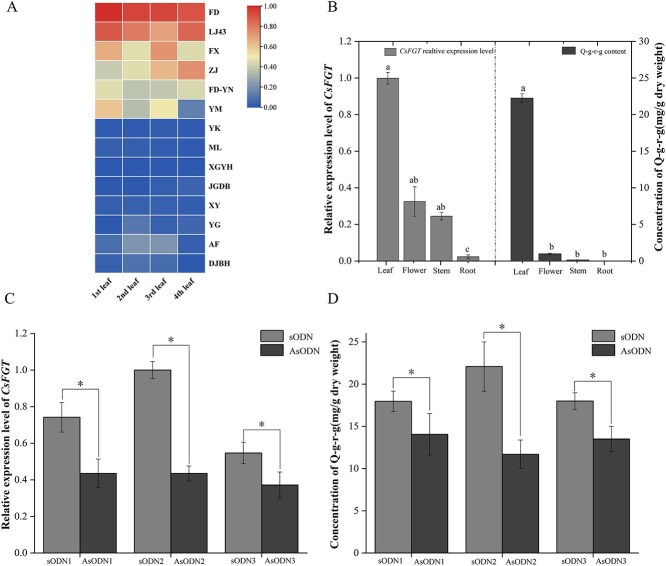
Expression pattern of *CsFGT* and its effect on Q-g-r-g content. (A) Relative expression level of *CsFGT* in leaves of various tea cultivars planted at the Tea Science Research Institute of Pu’er City, Yunnan Province (as listed in Supplementary Data Table S1). Normalized expression levels were visualized by using TBtools software and are indicated with different colors, and the raw data on expression levels are given in Supplementary Data Table S5. (B) Relative transcript abundance of *CsFGT* in different tissues of cultivars ‘Fudingdabaicha’ (FD) and ‘Longjing43’ (LJ43) planted in the experimental tea garden at the Tea Research Institute of Zhejiang University. (C and D) Effect of asODN injection on *CsFGT* expression (C) and Q-g-r-g content (D). Three asODNs or sODNs were used and designated asODN1/2/3 or sODN1/2/3. Oligonucleotide solutions (20 μM) were injected into tea leaves (500 μL g^−1^), then the injected leaves were immersed in oligonucleotide solutions and incubated at 25°C with 12 h light/12 h dark for 36 h; tea leaves treated with sODNs were used as control. After treatment, the leaves were subject to gene expression and Q-g-r-g content analysis. Each oligonucleotide treatment was conducted in three biological replicates. *Significant difference at *P* < 0.05.

The relationship between *CsFGT* expression level and Q-g-r-g content was studied in various tea cultivars of different varieties (Supplementary Data Table S1). The expression level of *CsFGT* in leaves of tea plants of the *sinensis* variety was significantly higher than that of leaves of the *assamica* variety, although the expression of *CsFGT* also varied with leaf position on the tender shoot (Fig. 7A and Supplementary Data Table S5). Meanwhile, a relatively high content of Q-g-r-g accumulated in leaves of cultivars of the *sinensis* type, but very low Q-g-r-g, even lower than the detection limit, was detected in leaves of many cultivars of the *assamica* type (Table 3). Analysis also showed that the expression level of *CsFGT* was significantly correlated with the content of Q-g-r-g (*r* = 0.878, *P* < 0.05) (Supplementary Data Fig. S6), indicating that the accumulation of Q-g-r-g in different cultivars was mainly regulated by expression level of *CsFGT*. Moreover, higher transcript levels of the gene were also observed in leaves and flowers rather than stems and roots, and were highly similar to the contents of Q-g-r-g in different tissues (Fig. 7B). It was clear that CsFGT can catalyse the conversion of Q-g-r-g from Q-g-r by exerting its glycosyl transferase activity.

**Table 3:** Content of Q-g-r-g in leaves of different cultivars (mg g^−1^ dry weight)^a^

Cultivar	Variety/parents	1st leaf^b^	2nd leaf^b^	3rd leaf^b^	4th leaf^b^	Average
FD	*sinensis*	19.77 ± 0.55	27.94 ± 0.20	22.17 ± 1.15	20.25 ± 0.49	22.53 ± 0.60
LJ43	*sinensis*	10.50 ± 0.27	9.27 ± 1.30	11.60 ± 0.20	10.17 ± 0.06	10.40 ± 0.50
JX	*sinensis*	2.45 ± 0.20	3.17 ± 0.06	2.22 ± 0.53	2.52 ± 0.03	2.59 ± 0.21
FX	FD (♀) × Yunkang 14 (♂)	3.06 ± 0.06	5.17 ± 0.45	5.52 ± 0.13	6.23 ± 0.47	5.00 ± 0.28
ZJ	*assamica*	2.94 ± 0.03	5.94 ± 0.06	5.67 ± 0.12	5.20 ± 0.26	4.94 ± 0.12
YM	*assamica*	0.36 ± 0.06	1.54 ± 0.31	2.08 ± 0.08	2.22 ± 0.09	1.55 ± 0.14
YK	*assamica*	0.00 ± 0.00	0.00 ± 0.00	0.00 ± 0.00	0.00 ± 0.00	0.00 ± 0.00
ML	*assamica*	0.00 ± 0.00	0.00 ± 0.00	0.00 ± 0.00	0.00 ± 0.00	0.00 ± 0.00
XGYH	*assamica*	0.00 ± 0.00	0.00 ± 0.00	0.00 ± 0.00	0.00 ± 0.00	0.00 ± 0.00
JGDB	*assamica*	0.00 ± 0.00	0.00 ± 0.00	0.00 ± 0.00	0.00 ± 0.00	0.00 ± 0.00
XY	*assamica*	0.00 ± 0.00	0.00 ± 0.00	0.00 ± 0.00	0.00 ± 0.00	0.00 ± 0.00
YG	*assamica*	0.00 ± 0.00	0.00 ± 0.00	0.00 ± 0.00	0.00 ± 0.00	0.00 ± 0.00
DJBH	*assamica*	0.00 ± 0.00	0.00 ± 0.00	0.00 ± 0.00	0.00 ± 0.00	0.00 ± 0.00

a All shoots (one bud and four leaves) with similar maturity were collected from the Tea Science Research Institute of Pu’er City, Yunnan Province. ^b^Leaf position down from the apical bud. Detailed information on the tea cultivars is given in Supplementary Data Table S1.

In order to confirm the function of *CsFGT in vivo*, three antisense oligodeoxyribonucleotides (asODNs) and sense oligodeoxyribonucleotides (sODNs) were injected into fresh tea leaves. Expression levels of *CsFGT* in asODN-treated leaves were significantly downregulated in comparison with sODN treatment (Fig. 7C). Meanwhile, Q-g-r-g contents in asODN-treated leaves were significantly lower than those in the sODN-treated ones. Comparatively, the variation in decrease of Q-g-r-g was smaller than that in downregulation of *CsFGT* in asODN-treated leaves (Fig. 7D). An explanation for this is that downregulated expression of *CsFGT* could inhibit new biosynthesis of Q-g-r-g, but could not influence the Q-g-r-g content that had been in the leaves before injection. The results indicated that biosynthesis of Q-g-r-g could be significantly inhibited when *CsFGT* was partially silenced by asODNs. Combined with the catalytic properties of the heterologously expressed protein and the relationship between *CsFGT* expression and Q-g-r-g content, *CsFGT* possesses glycosyl transferase activity and mainly contributes to Q-g-r-g biosynthesis in tea leaves.

## Discussion

### Function and significance of *CsFGT* in tea plant

**Figure 8:**
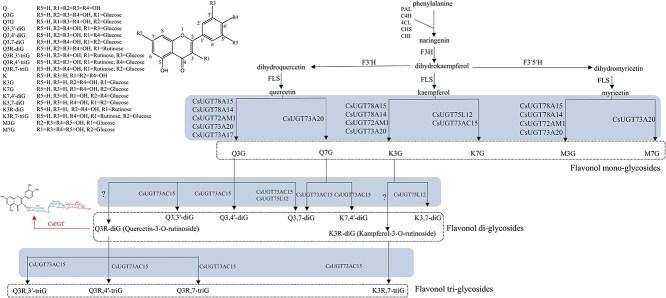
UGTs related to biosynthesis of the flavonol glycosides in tea plant*.* Most UGTs preferred to use UDP-glc as sugar donor. PAL, phenylalanine ammonia-lyase; C4H, cinnamate 4-hydroxylase; 4CL, 4-coumarate-CoA ligase; CHS, chalcone synthase; CHI, chalcone isomerase; F3H, flavanone 3-hydroxylase; F3′H, flavonoid 3′-hydroxylase; F3′5′H, flavonoid 3′,5′-hydroxylase; FLS, flavonol synthase.

Glycosylation of the small molecular compounds in plants is catalysed by subfamily 1 of GTs, also named UGTs, which exist widely and perform various functions in the plant kingdom [26]. Many UGTs have been screened from various plant species based on the conserved PSPG box. However, the functions of most UGTs have remained unclear due to their complex metabolic pathways and potential broad substrate spectra [23, 27, 28]. Among the 178 predicted *CsUGT*s of the tea plant, only a few genes have been experimentally identified. Among the identified ones, CsUGT73A17, CsUGT78A15, CsUGT78A14, CsUGT72AM1, CsUGT73A20, CsUGT75L12, CsUGT79B28, and CsUGT73AC15 have been proved to be involved in the synthesis of flavonol glycosides (Fig. 8) [5, 12, 14], while others could catalyse the glycosylation of volatile substances [29, 30]. Among the functionally identified genes involved in flavonol synthesis, most of them encode proteins that catalyse glycosylation towards flavonol to produce flavonol monoglycosides, but very few genes encode proteins catalysing the biosynthesis of flavonol diglycosides or flavonol triglycosides (Fig. 8); for example, CsUGT75L12 can catalyse the biosynthesis of quercetin 3-*O*,7-*O* diglycosides [13]. CsUGT73AC15 can transfer a glucosyl group to produce quercetin triglycosides, mainly onto the 7-OH position of the rutin, besides a few onto the 4′-/3′-OH [14]. In peach [16], soybean [17], *Fragaria × ananassa* [18], and *Pueraria lobata* [19], some UGTs were identified as enzymes catalysing the biosynthesis of flavonol diglycosides. So far, the gene involved in biosynthesizing flavonol 3-*O* triglycoside(s) from flavonol 3-*O* diglycoside(s) in tea plants has rarely been reported.

In this study, a *CsFGT* was screened out according to WGCNA analysis between content of flavonol triglycosides and transcription abundance of the DEGs in tender shoot leaves at various positions harvested from different representative cultivars. The ORF of the cloned *CsFGT* includes 1410 bp, and the deduced amino acid sequence possesses the highest homology with CaUGT3 (Fig. 1), able to mediate continuous transglycosylation to produce quercetin-3-*O*-gentiotetrioside [20]. Enzymatic characteristic tests showed that CsFGT can use Q-g-r and UDP-glc to produce Q-g-r-g, but also catalyse galactosyl transfer from UDP-gal to delphinidin to form delphinidin 3-*O* galactoside *in vitro* (Fig. 2), and can exert its highest catalytic efficiency at pH 8.0 and 30°C (Fig. 3A and B). It should be pointed out that the main function of CsFGT is to catalyse the biosynthesis of Q-g-r-g rather than delphinidin 3*-O* galactoside, since CsFGT exhibits much higher affinity and catalytic efficiency towards glucosylation of Q-g-r than towards galactosylation of delphinidin (Fig. 3C–F, Table 1). UGTs from various plant species, such as *Prunus persica*, *Punica granatum*, and *Pueraria lobata*, possess similar optimal reaction temperatures, but relatively different optimal pH values [31–33, 35]. According to our results, CsFGT exhibits relatively high catalytic efficiency in producing Q-g-r-g *in vitro.* The expression level of *CsFGT* in the leaves is highly correlated with the accumulation of Q-g-r-g among various cultivars. In asODN-treated leaves, the Q-g-r-g content significantly decreases along with downregulated expression of *CsFGT*. Based on this *in vitro* and *in vivo* evidence, *CsFGT* encodes an enzyme catalysing the biosynthesis of an important flavonol triglycoside, Q-g-r-g, which is greatly accumulated in the *sinensis* variety rather than the *assamica* variety.

Besides Q-g-r-g, there are other flavonol triglycosides in leaves of tea plant, such as quercetin 3-*O*-β-d-glucopyranosyl-(1→3)-α-l-rhamnosyl-dl-(1→6)-β-d-galactopyranoside (Q-ga-r-g), quercetin 3-*O*-α-l-rhamnopyranosyl-(1→3)-α-l- rhamnopyranosyl-(1→6)-β-d-galactopyranoside (Q-ga-r-r), quercetin 3-*O*-α-l- rhamnopyranosyl-(1→3)-α-l-rhamnopyranosyl-(1→6)-β-d-glucopyranoside (Q-g-r-r), kaempferol 3-*O*-β-d-glucopyranosyl- (1→3)-α-l-rhamnopyranosyl-(1→6)-β-d-glucopyranoside (K-g-r-g), kaempferol 3-*O*-β-d-glucopyranosyl-(1→3)-α-l-rhamnopyranosyl-(1→6)-β-d-galactopyranoside (K-ga-r-g), kaempferol 3-*O*-α-l-rhamnopyranosyl-(1→3)-α-l- rhamnopyranosyl-(1→6)-β-d-galactopyranoside (K-ga-r-r), and kaempferol 3-*O*-α-l-rhamnopyranosyl-(1→3)-α-l-rhamnopyranosyl-(1→6)-β-d-glucopyranoside (K-g-r-r) [15], indicating that many other UGTs might exist for producing the diverse flavonol triglycosides. These UGTs need to be further explored in the future from the large family of GTs through a precise screening strategy similar to that used this study in order to establish the synthesis pathway of the flavonol triglycosides.

### Potential key sites guarantee the catalytic specificity of CsFGT

In order to explain the catalytic specificity of CsFGT towards the sugar donor, we attempted to dock three sugar donors (UDP-glc, UDP-gal, and UDP- rha) with the enzyme when Q-g-r was used as a sugar acceptor. Residues of H17, P85, R140, G371, D391, and Q392 were all included in the interaction sites regardless of the type of sugar donor, indicating that these sites of CsFGT were responsible for recognition and interaction with the activated sugars (Fig. 4C–E). This finding was consistent with previous reports [35–37]. Meanwhile, a moiety of two successive amino acids (G371–S372) could simultaneously bind to the Q-g-r and the sugar donors. Interestingly, the three successive amino acid residues located in the loop region, G290, S291, and E292, could only interact with Q-g-r and UDP-glc to form a tight pocket favorable for glycosyl transfer, but not with Q-g-r and UDP-gal or Q-g-r and UDP-rha. It was clear that this moiety of three successive residues (G290–S291–E292) might be associated with the catalytic specificity of CsFGT towards Q-g-r and UDP-glc.

Furthermore, we tried to dock other sugar acceptors, including quercetin, kaempferol, and their monoglycosides, with CsFGT when UDP-glc was used as sugar donor. The predicted molecular orientation of quercetin or kaempferol was opposite to that of Q-g-r in the active pocket, which would prevent the activated glucosyl moiety from approaching the 3-OH on the C ring of quercetin or kaempferol in the spatial structure, and consequently hinder the formation of the Q-g or K-g (Fig. 4F and G). When flavonol monoglycoside (Q-g or K-g) was used as the acceptor, sugar moiety of the donor UDP-glc located at the edge of the docking pocket, and could hardly be attached to the 3-*O* site of the flavonol monoglycosides to form flavonol diglycosides, although the molecular orientation of the flavonol monoglycoside was similar to that of Q-g-r (Fig. 4H and I). When we attempted to dock K-g-r, another flavonol diglycoside similar to Q-g-r, and UDP-glc with CsFGT, the interaction pocket seemed much looser in comparison with that formed among the enzyme, Q-g-r, and UDP-glc (Fig. 4C and J), indicating that the number of hydroxyl groups on the B ring of the flavonol moiety would significantly impact the binding efficiency between CsFGT and substrates. A single hydroxyl group on the B-ring could hardly attract the amino acid residue, which would be unfavorable for forming an effective reaction pocket. Since CsFGT could catalyse the biosynthesis of delphinidin 3-*O* galactoside, docking was also performed between the enzyme and substrates (delphinidin and UDP-gal). Similar to the interaction among CsFGT, rutin, and UDP-glc, the successive amino acid residues G290, S291, and E292 in the loop domain, and Q352 in the PSPG box were also involved in reaction pocket formation among CsFGT, delphinidin, and UDP-gal, although Q352 participated in bringing the 3-OH of delphinidin closer to the galactosyl moiety of UDP-gal, instead of forming hydrogen bonds with the hydroxyl groups on the B ring and glycosyl of the rutin (Fig. 4C and K).

Site-directed mutagenesis is an important method to reveal the key catalytic and regulatory sites of the enzymes, and has also been proven to be efficient in the study of GT characteristics [38]. Catalytic activities were compared between wild-type CsFGT and mutants after candidate key sites had been predicted through molecular docking and mutated through site-directed codon replacement. Complete loss of glycosyl transfer activities was observed in mutants G290P, Q352S, Q352E, and G290P–S291T–E292T, while partial loss was seen in mutants E292T, Q352A, and Q352T (Fig. 5B and C). Clearly, enzymatic activity would be greatly influenced by mutation of Q352, which was located in the PSPG box of CsFGT. The PSPG box was regarded as the key region for recognition of UDP-sugar [35, 39]. The amino acid residue mutation E396A in the PSPG box of TcCGT1 abolished the catalytic activity because of a recognition defect of UDP-sugar [40]. The residue D382 of UGT708C1 in buckwheat (*Fagopyrum esculentum*) was identified as a key site in recognizing the sugar moiety of the donor, and mutants D382N and D382A almost lost their activities completely [41]. Residues E352 and Q353 of the macrolide glycosyltransferase in *Escherichia coli* GT-28 were predicted as the key sites to interact with the sugar donor; activities of the E352A and Q353A mutants were 425- and 106-fold lower than that of the wild-type enzyme [42]. However, according to our molecular docking results, activity loss of the Q352 mutation to other amino acids was mainly due to weakened association between the enzyme and the hydroxyl groups of rutin (sugar acceptor) instead of the UDP-sugar (sugar donor) (Fig. 6I–L). Recent reports confirmed that mutation of some residues in the PSPG box would significantly impact the binding of the sugar acceptor and regioselectivity of UGTs [43, 44]. Obviously, the PSPG box could directly interact with the sugar donor and/or the sugar acceptor and indirectly influence the spatial structure of the enzyme, which is crucial for the catalytic attributes of the UGTs . Meanwhile, catalytic activity of CsFGT could also be greatly influenced by single mutations (G290P, E292T, and R319N) outside the PSPG box (Fig. 6B, E, and G), which is in line with many other reports [45–47].

It was interesting that three successive amino acid residues (G290, S291, and E292) were predicted to locate in the loop region and be able to simultaneously interact with the sugar donor and acceptor. The triple mutation G290P–S291G–E292T would lead to complete loss of enzyme activity since the loop region of the mutant became loose and could not efficiently connect the sugar acceptor and donor together (Fig. 6M and N). This finding was consistent with the previous observation that the loop region could help to bring the sugar donor and acceptor closer [25]. In some previously published papers, mutants harboring residue mutations in the loop region of GT could recognize and use new substrates to generate novel products [25, 48, 49]. Unfortunately, the G290P–S291G–E292T mutant could not recognize and catalyse other substrates except Q-g-r and UDP-glc, although we also attempted to use kaempferol, quercetin, K-g, Q-g, and K-g-r as sugar acceptors (Supplementary Data Fig. S7). This implied that UGTs from different plants may possess their own special characteristics besides the shared properties of their family. Collectively, the amino acid residues of G290, R319, and Q352 could bind to the two hydroxyl groups on the B ring of Q-g-r and played crucial roles in the formation of the active reaction pocket. Additionally, the three successive amino acid residues (G290, S291, and E292) interacted with both the sugar donor and acceptor, facilitating their favorable spatial orientation for catalysis.Report showed that CsUGT73AC15 could catalyse glucosylation of rutin and kaempferol 3-*O*-rutinoside at 7-OH position to produce flavonol triglycoside [14]. However, there were significant differences in spatial structure and molecular docking of CsUGT73AC15 and CsFGT (Supplementary Data Fig. S8A and B and Fig. 4A and C). CsUGT73AC15 seemed to form an active pocket favorable to catalysis of the glucosylation at 7-OH of rutin and kaempferol 3-*O*-rutinoside instead of at the 3-*O*-rutinosyl group (Supplementary Data Fig. S8C and D). Sequence alignment showed that CsUGT73AC15 only shared around 27% identity with CsFGT and lacked many key amino acid residues identified in CsFGT (Supplementary Data Fig. S8E).

### Expression and regulation of *CsFGT* in different tea germplasms

Catechins and flavonol glycosides are the two main types of phenolic substances in the leaves of all tea plants [50], but their compositions in *sinensis* and *assamica* varieties are quite distinctive; in particular the *assamica* variety tends to accumulate ECG and flavonol monoglycosides, while *sinensis* prefers to have a high content of EGCG and flavonol di- and triglycosides [15, 51]. Therefore, the number of hydroxyl groups at the B-ring of catechins and the sugar chain length at the C-ring of flavonol glycosides has been recommended to distinguish the two varieties. Why do the two varieties exhibit an obvious difference in the trait of phenolic composition? According to recent genomic studies, a divergence event between *sinensis* and *assamica* varieties might have taken place around 0.38–1.54 million years ago [52], after which the two varieties followed their independent evolutionary histories and parallel domestication although extensive intra- and interspecific introgressions, which often occurred [53], and selection for disease resistance and flavor in *sinensis* populations seemed stronger than that in *assamica* populations during their domestication [22]. The composition difference of catechins and flavonol glycosides might reflect the evolutionary diversity of the two varieties during migration and domestication. Research revealed that a 14-bp deletion at the upstream regulatory domain of the *F3′5′H* promoter, leading to low expression of *F3′5′H*, was closely related to low production of catechins with trihydroxyl groups at the B-ring in the *assamica* variety [50]; in contrast, a promoter with the 14-bp domain would activate *F3′5′H* expression and enhance the accumulation of catechins with trihydroxyl groups at the B-ring in the *sinensis* variety [22].

A report showed that the accumulation patterns of flavonol glycosides differed significantly in *sinensis* and *assamica* varieties, and the leaves of the *sinensis* variety tended to accumulate many more flavonol triglycosides, like Q-g-r-g, compared with the *assamica* variety [15]. Studies showed that the composition and content of flavonol glycosides were mainly related to plant species, varieties and germplasms, tissue parts, development stages, and growth environments [55]. Significant progress has been made in identifying the key enzymes and regulators involved in the accumulation of flavonol glycosides in model plants and horticultural plants [56–60]. In tea plant, CsMYB12 and CsbZIP1 could activate the expression of *CsFLS* and *CsUGT78A14*, respectively, through interaction with their promoters, leading to increased accumulation of flavonol monoglycosides under high light conditions or UV radiation [61]. According to our findings in this study, CsFGT could efficiently catalyse Q-g-r-g formation and significantly influence the content of flavonol triglycosides in cultivars belonging to different varieties through its differential expression (Supplementary Data Fig. S6). Extremely high similarities were observed in CsFGT amino acid sequences obtained from different cultivars belonging to the *sinensis* and *assamica* varieties (Supplementary Data Fig. S2); thus, synthesis of flavonol triglycosides might be differentially and strictly regulated at transcriptional level of the relevant key gene(s), and the differential expression of *CsFGT* in the two varieties might be the main reason for their differential accumulation of Q-g-r-g. Different insertions/deletions/mutations might have been present in the *CsFGT* promoter during parallel domestication of the two varieties, which would influence the interaction between promoter and the relevant transcription factor(s). Similar situations might also occur in the differential accumulation of other flavonol triglycosides, such as Q-ga-r-g, Q-ga-r-r, Q-g-r-r, K-g-r-g, K-ga-r-g, K-ga-r-r, and K-g-r-r. Therefore, studies on the expression regulation of *CsFGT* and other genes involved in the flavonol triglycoside biosynthesis pathway will provide a significant clue about genetic evolution among different varieties. However, the differential accumulation mechanism of flavonol glycosides may be complex and needs to be comprehensively studied in the future.

## Materials and methods

### Plant materials and chemicals

A representative cultivar, FD (5 years old), of the *C. sinensis* (L.) O. Kuntze var. *sinensis* used in this study for gene separation was cultivated in the experimental tea garden of the Tea Research Institute of Zhejiang University. Tender leaves were picked up and immediately frozen in liquid nitrogen and stored at −80°C until use. In order to analyse gene expression and flavonol glycosides, the first to fourth leaves down from the apical bud were separately harvested from tender shoots of different tea cultivars (14 years old) planted at Pu’er City, Yunnan Province, and detailed information about these cultivars is listed in Supplementary Data Table S1. Different tissues (leaf, young root, tender stem, and flower) were harvested from FD and ‘Longjing 43’ (LJ43, 5 years old) from the experimental tea garden of the Tea Research Institute of Zhejiang University. The harvested fresh samples were divided into two parts; one part was frozen in liquid nitrogen and stored at −80°C for subsequent gene expression analysis, and the other part was denatured in a microwave oven at 80 W for 2 min, then dried at 80°C for 4 h and used for flavonol glycoside analysis by UPLC–DAD–MS/MS. All samples were prepared in triplicate.

Reference compound Q-g-r-g was obtained by preparative chromatography separation as described previously [15]. Q-g-r was purchased from Aladdin (Shanghai, China). Quercetin, kaempferol, Q-g, K-g, kaempferol-3-*O*-galactoside (K-ga), K-g-r, delphinidin, cyanidin-3-*O*-galactoside, cyanidin-3,5,-*O-*diglucoside, pelargonidin, malvidin, (−)epicatechin (EC), (−)epigallocatechin gallate (EGCG), (−)epicatechin gallate (ECG), (+)gallocatechin (GC), and (−)epigallocatechin (EGC) were obtained from DeSiTe Biological Technology Co., Ltd (Chengdu, China). UDP-rha, UDP-glc, and UDP-gal were purchased from Macklin (Shanghai, China). HPLC-grade acetonitrile, formic acid, acetic acid, methanol, and ethanol were ordered from Merck Co. (Shanghai, China). The highly pure water used throughout the experiment was prepared using the Barnstead™ GenPure™ water system (ThermoFisher Scientific, USA). All other chemicals used were of analytical grade.

### Gene cloning and bioinformatics analysis

According to our previous WGCNA analysis on the relationship between expression levels of the DEGs and contents of the various flavonol glycosides in the leaves of the representative cultivars FD (belonging to variety *sinensis*) and YK (variety *assamica*) (Supplementary Data Fig. S1), five differentially expressed *UGT* candidates possibly catalysing the biosynthesis of the flavonol triglycoside were screened out. Primer pairs (Supplementary Data Table S2) for cloning the ORF of these five genes were designed according to the homologs in the published LJ43 genomic data [22]. These genes were then cloned from tea cultivars FD and YK by RT–PCR, but only one gene-encoded protein could catalyse biosynthesis of flavonol triglycoside and was named *CsFGT*. The gene was cloned and sequenced according to the following procedure. Total RNA was isolated from the collected leaves of FD and reverse-transcribed into cDNAs. The ORF of *CsFGT* was obtained after RT–PCR amplification with the cDNAs and gene-specific primers (Supplementary Data Table S2). The target band of the ORF was purified and ligated into pMD18-T vector (Takara, Dalian, China). The recombined T vector (pMD18-CsFGT) was transformed into DH5α-competent cells and sequenced by Sangon Biotech Technology Co., Ltd (Shanghai, China). The amino acid sequence was deduced from the obtained ORF using software EditSeq v7.1.0 (DNASTAR, Inc., WI, USA), and used to analyse the bioinformatic characteristics. Phylogenetic analysis was performed using MEGA11 (https://www.megasoftwaretree nodes .net), and evolutionary distances were estimated by a p-distance method and evaluated with a bootstrap value of 1000. Sequence alignment of the *UGT*s was carried out with Jalview version 2.11.3.2 (The Barton Group, University of Dundee) using the Muscle method with default sets and embellished using Gendoc (https://github.com/karlnicholas/GeneDoc). Protein secondary structure was predicted using SOPMA software (https://npsa-prabi.ibcp.fr/cgi-bin/npsa_automat.pl?page=/NPSA/npsa_sopma.html), and transmembrane regions were predicted using the TMHMM server (https://services.healthtech.dtu.dk/service.php?TMHMM-2.0). The relative molecular weight and theoretical isoelectric point of CsFGT were analysed using the ExPASy-ProtParam online tool (https://web.expasy.org/protparam/) and conserved domain analysis was performed on NCBI (https://www.ncbi.nlm.nih.gov/Structure/cdd/wrpsb.cgi).

### Subcellular localization

The *CsFGT* ORF without the stop codon was amplified by PCR and then cloned into the expression vector pCAMBIA1304:GFP under the control of the CaMV 35S promoter by homologous recombination. The clone primers are listed in Supplementary Data Table S2. The recombinant vectors were transformed into *Agrobacterium tumefaciens* GV3101-competent cells, and then were injected into tobacco leaves and onion inner epidermis using the *Agrobacterium*-mediated method. After 48 h of infiltration, the tobacco leaves were collected and examined with a confocal laser scanning electron microscope (Olympus, FV3000, Japan). mCherry-PM was used as a cell membrane localization marker in tobacco leaf cells and DiD (1,1′-dioctadecyl-3,3,3′,3′-tetramethylindodicarbocyanine,4-chlorobenzenesulfonate salt) was used as a far-red plasma membrane fluorescent dye for onion epidermal cells.

### Heterologous expression of *CsFGT*

The coding sequence of *CsFGT* was amplified from DH5α-containing recombined pMD18-CsFGT with gene-specific primers harboring the homologous sequence of the expression vector at the 5′ end (Supplementary Data Table S2). The target product was purified and cloned into a linearized pCold-TF vector embedded with a 6× His-tag (Miaoling Biotech Co., Wuhan, China) using a Homologous Recombination Kit (Vazyme Biotech Co., Nanjing, China), and the recombinant plasmid (pCold-TF-CsFGT) was transformed into Origami (DE3)-competent cells (2nd Lab, Shanghai, China) after sequence confirmation. The transformed cells were amplified, then induced at 16°C for 24 h after addition of 1 mM IPTG, and then disrupted by lysozyme. The soluble recombinant protein (rCsFGT) was obtained after removal of cell debris by centrifugation at 5000 × g for 10 min and purified on a nickel ion purification column embedded with His-tag affinity resins (MCE, Shanghai, China). To remove the tag, the purified recombinant protein (100 μg) was mixed with 1 μg HRV3C protease in reaction buffer (25 mM Tris–HCl, pH 7.0, 150 mM NaCl, 0.5 mM EDTA, and 1 mM DTT), and incubated at 4°C for 16 h. The trigger factor (TF)-removed target protein (CsFGT) was obtained after the digestive products had passed the nickel ion purification column and confirmed by 10% SDS–PAGE. The empty plasmid pCold-TF without exogenous gene insertion was also transformed into DE3 cells, amplified and induced to express as described above, and the expressed protein was also purified by affinity chromatography and used as control protein during enzymatic activity assays. Protein concentration was quantified with a BCA Protein Quantification Kit (Yeasen, Shanghai, China).

### Analysis of substrate specificity and kinetic parameters

To analyse the relative catalytic activity of the purified recombinant protein rCsFGT and the TF-removed protein CsFGT towards different substrates, a 200-μL reaction mixture was prepared, consisting of 100 μM/160 μM sugar acceptor, 2 mM sugar donor (UDP-rha/UDP-glc/UDP-gal), 10 μg purified target protein or control protein, and 50 mM Tris–HCl buffer (pH 7.5). The sugar acceptors, including flavonols and glycosides (kaempferol, K-g, K-ga, K-g-r, quercetin, Q-g, Q-g-r), anthocyanins and glycosides (delphinidin, cyanidin-3-*O-*galactoside, cyanidin-3,5,-*O*-diglucoside, pelargonidin, malvidin), and catechins (EC, EGCG, ECG, GC, EGC), were tested one by one. Each reaction mixture was incubated at 30°C for 60 min and then heated at 100°C for 10 min to denature the enzyme. The heated reaction mixture was centrifuged at 12 000 × g and 4°C for 10 min and the supernatant was used to analyse the content of substrates and products by HPLC or UPLC–DAD–MS/MS.

Tests for optimal reaction acidity and temperature were carried out after the dominant sugar donor and acceptor had been obtained. The optimal reaction acidity of the rCsFGT was determined at 30°C for 60 min by serial testing in which a variety of buffers were applied, including 50 mM citrate buffer (buffer 1, pH 3.0–6.0), 50 mM Tris–HCl buffer (buffer 2, pH 5.0–8.0), and 50 mM phosphate buffer (buffer 3, pH 7.0–10.0). To determine the optimal reaction temperature of rCsFGT, the reaction was performed at a series of temperatures (from 5°C to 60°C with a 5°C increment) for 60 min. After the reaction, heating and centrifugation were carried out as above to denature and remove the enzymes. The supernatant was used to analyse the flavonol glycosides by HPLC.

Tests for determining the *K*_m_ and V_max_ of rCsFGT towards UDP-glc and rutin were conducted at the optimal acidity and temperature for 10 min using different levels of sugar donor (0, 20, 40, 60, 80, 100, 150, 200, 400, 600, 800, and 1000 μM) and sugar acceptor (0, 2, 4, 6, 8, 10, 20, 30, 40, 50, 75, 100, 150 and 200 μM) as substrates. Tests for determining the *K*_m_ and V_max_ of rCsFGT towards UDP-gal and delphinidin were conducted for 10 min using different levels of sugar donor (0, 200, 400, 600, 800, 1000, 1200, 1400, 1600, 1800, and 2000 μM) and sugar acceptor (0, 200, 400, 600, 800, 1000, 1200, 1400, 1600, 1800, and 2000 μM) as substrates. The concentration of the product UDP was used to quantify the enzymatic activity and measured according to the UDP-Glo™ Glycosyltransferase Assay instructions (Promega, Beijing, China). Kinetic parameters were calculated by fitting the test data to the Michaelis–Menten equation (1/*V* = *K*_m_/*V*_max_ × 1/*S* + 1/*V*_max_) using Origin 2021 software.

### Expression suppression of *CsFGT* by asODN injection

Candidate sequences of asODNs (Supplementary Data Table S2) were selected using RNAfold online software (https://sfold.wadsworth.org/cgi-bin/soligo.pl), and thiophosphorylations were modified onto the last three nucleotides at the end of asODNs to improve stability. The sODNs were also designed as control. AsODNs and sODNs were synthesized by Sangon Biotech Co., Ltd (Shanghai, China). Oligonucleotide solutions (20 μM) were prepared and injected into tea leaves (500 μL g^−1^) using a sterile syringe, and the injected leaves were immersed in 1 mL oligonucleotide solution and incubated at 25°C and 12 h light (1500 lux)/12 h dark for 36 h. Three biological replicates were carried out for each oligonucleotide. After incubation, a small portion of the leaves was sampled from each treatment and used for gene expression analysis, and the remaining leaves of each treatment were washed with water twice and denatured by microwave heating at 80 W for 1 min, and dried at 80°C for 1 h and used for UPLC–DAD–MS/MS analysis.

### Analysis of flavonol glycosides, anthocyanins, and catechins

Analysis of the flavonol glycosides in the enzymatic reaction mixture was performed on an HPLC (Shimadzu Co., Kyoto, Japan) equipped with a Zorbax 5 μm TC-C_18_(2) chromatographic column (250 × 4.6 mm, Agilent Technologies Inc., CA, USA). The HPLC conditions were as follows: oven temperature, 33°C; injection volume, 10 μL; mobile phase A, acetonitrile/acetic acid/water = 3/0.5/96.5 (v/v/v); mobile phase B, acetonitrile; gradient elution, linearly increasing phase B from 8% to 30% in the first 10 min, then to 60% in the next 10 min, and holding phase B at 60% for 15 min, finally returning phase B to 8% in 10 min; detection wavelength, 350 nm. Flavonol glycosides were identified and quantified by comparing the retention time and peak area with reference compounds [15]. Reaction products after catalysis of CsFGT were separated by HPLC and confirmed by NMR. The product in the eluate was collected after HPLC separation and pooled, and then freeze-dried in a JGL-10 vacuum freeze dryer (Beijing Songyuan Huaxing Technology Development Co., Ltd, China) and finally re-suspended in 0.6 mL CD_3_OD for ^1^H- and ^13^C-NMR measurements. The spectra were recorded on a Bruker Avance III spectrometer at 500 MHz with a 5-mm DC hyperpolarized cryoprobe (Bruker, Wissembourg, France). Integration of the spectra was performed using Mestnova 9.0.1 (Mestrelab Research S.L., Santiago de Compostela, Spain).

Flavonol glycosides in the leaves of different tea cultivars (Supplementary Data Table S1) and asODN injection tests were extracted with 50% aqueous ethanol and detected on an UPLC–DAD–MS/MS (Waters Co. Ltd, Milford, MA, USA) equipped with a Waters CORTECS T3 (2.1 × 100 mm, 1.6 μm) column as described in our previous paper [15]. Flavonol glycosides were identified based on comparing the retention time and ion signals with reference compounds, and quantified according to the peak area at 350 nm. The product Q-g-r-g in the enzymatic reaction was also confirmed using UPLC–DAD–MS/MS by DAD scan and MS daughter scan. The DAD scan was conducted from 190 to 400 nm, and daughter signals of the parent ion [M-H]^−^ of 771 *m*/*z* were scanned with collision gas flow of 0.13 mL min^−1^ and collision voltage of 20 V in negative mode.

Detection of anthocyanins and catechins in the reaction solution was performed by HPLC and UPLC–DAD–MS, and detailed performance was carried out according to methods described previously [15, 24].

### Analysis of *CsFGT* expression

Total RNAs were extracted from the leaves of various tea cultivars, different tissues of FD and LJ43, and asODN-treated leaves, then reverse-transcribed into cDNAs as described above. Analysis of *CsFGT* expression was carried out with gene-specific primers (Supplementary Data Table S2) on a StepOnePlus™ Real-Time PCR System (Applied Biosystems) according to the previously described method [62]. The housekeeping gene *β-actin* was chosen as the reference in order to normalize transcription abundance, and relative gene expression was calculated using the 2^−ΔΔCT^ method after checking the melting curve.

### Protein homology modeling, molecular docking, and site-directed mutagenesis

The 3D structure of CsFGT was predicted by the comparative modeling method on the SWISS-MODEL server (https://swissmodel.expasy.org/) using the crystal structure of a rice glycosyltransferase protein (SMTL ID 7es0.1, Protein Data Bank No. Q0DPB7) as the template. To evaluate the stereochemical quality of the predicted CsFGT structure, the Ramachandran map was also generated using PROCHECK (https://www.ebi.ac.uk/thornton-srv/software/PROCHECK/index.html). The 3D structures of the UDP-sugars (UDP-glc, CID 8629; UDP-gal, CID 18068; UDP-rha, CID 192751), flavonols and their glycosides (quercetin, CID 5280373; kaempferol, CID 5280863; Q-g, CID 5280804; K-g, CID 52802102; Q-g-r, CID 5280805; K-g-r, CID 5318767) as well as delphinidin (CID 128853) were obtained from PubChem (https://pubmed.ncbi.nlm.nih.gov). The potential interaction among CsFGT, sugar donor and acceptor was investigated using molecular docking software in AutoDockTools Vina 1.5.7 [63]. CsFGT protein was set to the center position, and the docking box size was set as 30 × 30 × 30 grid points (each grid point representing a distance of 1.000 nm). Molecular docking efficiency was evaluated according to the Auto-Grid lowest energy. Model skeleton structure between CsFGT and substrates was visualized by the Python script. The key amino acid scan was performed using FoldX software [64]. The RMSF value was calculated using YASARA software [65].

A series of site-directed mutations were generated through a site-directed mutagenesis kit (Vazyme, Nanjing, China) using the pCold–TF–CsFGT plasmid as the template and codon-replaced oligonucleotides as special primer pairs (Supplementary Data Table S2). PCR amplification was performed according to the following program: denaturing at 98°C for 30 s, 1 cycle; denaturing at 98°C for 15 s, annealing at 60°C for 15 s, extension at 72°C for 7 min, 30 cycles; and final extension at 72°C for 5 min. Site-directed pCold–TF–CsFGT mutant was transformed into DH5α for amplification and mutations were verified by Sanger sequencing (Sangon Biotech, Shanghai). The amplified mutant of pCold–TF–CsFGT was extracted and transferred into DE3 to express the mutated proteins. The mutated protein was induced and extracted, then purified using His-tag affinity resins and checked by SDS–PAGE as described above. The purified mutant protein (single amino acid replacement) was used to examine enzyme activity towards Q-g-r and UDP-glc accordingly. For the successive three amino acid mutant G290P–S291G–E292T, enzyme activity was detected using different sugar acceptors (kaempferol, quercetin, K-g, Q-g, and K-g-r) and donors (UDP-gal, UDP-rha, and UDP-glc) besides Q-g-r and UDP-glc. The reaction was performed and the product was monitored as described above. The heterologously expressed wild rCsFGT was used as control.

### Statistical analysis

Analysis of statistics was performed on IBM SPSS Statistics for Windows version 26.0 (IBM Corp., Armonk, N., USA) using a one-way ANOVA model.

## Supplementary Material

Web_Material_uhaf149

## Data Availability

All data related to this research are available in this paper and its supplementary materials published online. GenBank accession numbers involved in this article are as follows: Phylogenetic tree of plant UGTs: CsFGT (OR487152.1); AtUGT73C3 (NP_181217.1); CsUGT703B1 (KJ381079.1); AtUGT76E5 (Q9STE6.1); CsUGT75L12 (ALO19892.1); Cp3GT (ACS15351); Cs3GT (AAS00612.2); CsUGT73A20 (ALO19886.1); AtUGT78D3 (OAO94865.1); PgUGT95B2 (AZB52139.1); Sb3GT1 (QBL54224.1); PoUGT72B11 (ACB56923.1); CsUGT707B1 (CCG85331.1); VvGT5 (BAI22846.1); CsGT45 (ACM66950.1); CaUGT3 (BAH80312.1); GmUGT73C20 (XP_003518710); PgUGT94Q4 (QEA68984.1); PgUGT73A18 (QEA68968.1); PgUGT74T4 (QEA68972.1); PgUGT75W1 (QEA68973.1); CsUGT73A17 (BAO51837.1); VvGT1 (NP_001384786.1); AtUGT78D1 (OAP13716.1); MdUGT71A15 (NP_001315903.1); AtUGT72D2 (OAO89857.1); SsGT1 (AY033489); CcUGT703H1 (QNT13160.1); CcUGT729A2 (QNT13161.1); AmUGT73E2 (BAG16513.1); PfUGT88A7 (BAG31949.1); AmUGT73N1 (BAG16514.1); SiUGT88D6 (BAG31947.1); PfUGT88D7 (BAG31948.1); PfUGT73A7 (BAG31951.1); CsUGT72AM1 (ASA40331.1); Gt5GT7 (B2NID7.1); NtGT2 (BAB88935.1); AmUGT73A9 (BAG31950.1); AmUGT88D4 (BAG31945.1).
