# Supplementary material for: Functional analysis of a UDP-glucosyltransferase gene contributing to biosynthesis of the flavonol triglycoside in tea plants
Source: Hortic Res. 2025 May 6;12(9):uhaf149. doi: 10.1093/hr/uhaf149 (PMC12313340; doi:10.1093/hr/uhaf149)
Supplement: Web_Material_uhaf149 [file web_material_uhaf149.zip › Supplementary files (revised).pdf]

Supplementary files

Table S1. The detailed information of tea plant cultivars*.

No.	Cultivar	Plantation location	Leaf size	Variety/parents	Abbreviation
1	Fudingdabaicha	Hangzhou, Zhejiang<br>Pu'er, Yunnan	Medium	*sinensis*	FD
2	Jinxuan	Pu'er, Yunnan	Medium	*sinensis*	JX
3	Longjing 43	Hangzhou, Zhejiang<br>Pu'er, Yunnan	Medium	*sinensis*	LJ43
4	Foxiang	Pu'er, Yunnan	Large	Fudingdabaicha (♀)<br>×Yunkang 14 (♂)	FX
5	Duanjiebaihao	Pu'er, Yunnan	Large	*assamica*	DJBH
6	Jinggudabaicha	Pu'er, Yunnan	Large	*assamica*	JGDB
7	Mulan 1#	Pu'er, Yunnan	Large	*assamica*	ML
8	Xiangguiyinhao	Pu'er, Yunnan	Large	*assamica*	XGYH
9	Xueya 100	Pu'er, Yunnan	Large	*assamica*	XY
10	Yungui	Pu'er, Yunnan	Large	*assamica*	YG
11	Yunkang 10#	Pu'er, Yunnan	Large	*assamica*	YK
12	Yunmei	Pu'er, Yunnan	Large	*assamica*	YM
13	Zijuan	Pu'er, Yunnan	Large	*assamica*	ZJ

*. Tender leaves were plucked from different cultivars and divided into two parts, one was used for gene cloning and expression in which the leaves were immediately frozen with liquid nitrogen and stored at -80 °C, another was used for chemical analysis in which the leaves were denatured in a microwave oven at 80 W for 2 min, then dried at 80 °C for 4 h.

Table S2. The information of the primer pairs and oligonucleotides*.

Purpose	Gene name	Primer name	Sequences (5'→3')
Cloning	*CsFGT*	F	ATGGAGAGAGACATTCACGCTG
		R	TCACTTCCCATTTTTCAGATACTCAAC
	evm.model.Cha0	F	ATGAAGACAGAGACTCACGTCG
	6g008370	R	TTAGACACCCAAACCTGACTTCTC
	evm.model.Cha0	F	ATGGCTAAGAAAGATCAAAAGCTCAGT
	4g001710	R	CTATTTTTGAAGAGCATAAGCTTTG
	evm.model.Cha1	F	ATGGCCACATGCCACGTGGT
	1g008220	R	TCATTTGGAGATATTCTTGA
	evm.model.Cha0	F	ATGGCTAAGGAAGATCAAA
	4g003460	R	TTATTTTTGAAGAGCATAAGC
qPCR	*CsFGT*	F	CCGGTAGAGGCAAAGTCTCA
		R	AGAGGCAAAACAACCATACAATG
	Actin	F	CTTCCTCATGCTATCCTCCGTCTT
		R	ATTTCCCGTTCAGCAGTGGTG
pCold-TF fusion protein expression	*CsFGT*	F	ggtaggcatatggagATGGAGAGAGACATTCACGCTG
		R	ctcgagggtaccgagTCACTTCCCATTTTTCAGATACTCAAC
	evm.model.Cha0	F	ggtaggcatatggagATGAAGACAGAGACTCACGTCG
	6g008370	R	ctcgagggtaccgagTTAGACACCCAAACCTGACTTCTC
	evm.model.Cha0	F	ggtaggcatatggagATGGCTAAGAAAGATCAAAAGCTCAGT
	4g001710	R	ctcgagggtaccgagCTATTTTTGAAGAGCATAAGCTTTG
	evm.model.Cha1	F	ggtaggcatatggagATGGCCACATGCCACGTGGT
	1g008220	R	ctcgagggtaccgagTCATTTGGAGATATTCTTGA
	evm.model.Cha0	F	ggtaggcatatggagATGGCTAAGGAAGATCAAA
	4g003460	R	ctcgagggtaccgagTTATTTTTGAAGAGCATAAGC
Subcellular localization	*CsFGT*	F	ggactcttgaccatgATGGAGAGAGACATTCACGCTG
		R	agtcagatctaccatCTTCCCATTTTTCAGATACTCAAC
Gene suppression	*CsFGT*	sODN1	TTTGTCGCATTCGGCCACTT
		AsODN1	AAGTGGCCGAATGCGACAAA
		sODN2	TTCGGCCACTTAATTCCATT
		AsODN2	AATGGAATTAAGTGGCCGAA
		sODN3	CACCCGATTGGATCATCTCC
		AsODN3	GGAGATGATCCAATCGGGTG
Site-directed mutation	G290A	F	TTCGCGAGCGAGTGTAAATTCTCCAGAGACCA
		R	TTACACTCGCTCGCGAACCCAACAAACACCACGG
	G290P	F	GTTCCCAAGCGAGTGTAAATTCTCCAGAGACC
		R	TACACTCGCTTGGGAACCCAACAAACACCACGG
	S291G	F	GTTCGGAGGAGAGTGTAAATTCTCCAGAGACCAAGTT
		R	TACACTCTCCTCCGAACCCAACAAACACCACG
	E292Q	F	GTTCGGAAGCCAATGTAAATTCTCCAGAGACCAAGTT
		R	TACATTGGCTTCCGAACCCAACAAACACCACG
	E292T	F	CGGAAGCACGTGTAAATTCTCCAGAGACCAAGTTTAC
		R	ATTTACACGTGCTTCCGAACCCAACAAACACC
	Triple mutants	F	GTTCCCAGGAACGTGTAAATTCTCCAGAGACCAAGTTTAC
		R	TACACGTTCCTGGGAACCCAACAAACACCACGG
	R319N	F	GGCTCTGAACAAACCCAGTTGGGCTTCCGACG
		R	TGGGTTTGTTCAGAGCCCACAAAAATGGCAGT
	R319P	F	TCTGCCGAAACCCAGTTGGGCTTCCGACGATC
		R	AACTGGGTTTCGGCAGAGCCCACAAAAATGGCA
	Q352A	F	CACCGGCGATCGAGATTCTGGCTCACCCATCA
		R	AATCTCGATCGCCGGTGCCCATCCGATTGAGA
	Q352S	F	CACCGAGCATCGAGATTCTGGCTCACCCATCA
		R	AATCTCGATGCTCGGTGCCCATCCGATTGAGA
	Q352E	F	CACCGGAGATCGAGATTCTGGCTCACCCATCA
		R	AATCTCGATCTCCGGTGCCCATCCGATTGAGA
	Q352I	F	GGCACCGATAATCGAGATTCTGGCTCACCCAT
		R	TCTCGATTATCGGTGCCCATCCGATTGAGACT

*. Lowercase base represented the terminal homologous sequence against the vector; and underlined base indicated thiophosphorylation-modified nucleotide for stability improvement; base with red font represented the codon of the mutation site.

Table S3. The GenBank accession number of UGTs used in the phylogenetic analysis.

Gene names	Accession number
CsFGT	OR487152.1
AtUGT73C3	NP_181217.1
CsUGT703B1	KJ381079.1
AtUGT76E5	Q9STE6.1
CsUGT75L12	ALO19892.1
Cp3GT	ACS15351
Cs3GT	AAS00612.2
CsUGT73A20	ALO19886.1
AtUGT78D3	OAO94865.1
PgUGT95B2	AZB52139.1
Sb3GT1	QBL54224.1
PoUGT72B11	ACB56923.1
CsUGT707B1	CCG85331.1
VvGT5	BAI22846.1
CsGT45	ACM66950.1
CaUGT3	BAH80312.1
GmUGT73C20	XP_003518710
PgUGT94Q4	QEA68984.1
PgUGT73A18	QEA68968.1
PgUGT74T4	QEA68972.1
PgUGT75W1	QEA68973.1
CsUGT73A17	BAO51837.1
VvGT1	NP_001384786.1
AtUGT78D1	OAP13716.1
MdUGT71A15	NP_001315903.1
AtUGT72D2	OAO89857.1
SsGT1	AY033489
CcUGT703H1	QNT13160.1
CcUGT729A2	QNT13161.1
AmUGT73E2	BAG16513.1
PfUGT88A7	BAG31949.1
AmUGT73N1	BAG16514.1
SiUGT88D6	BAG31947.1
PfUGT88D7	BAG31948.1
PfUGT73A7	BAG31951.1
CsUGT72AM1	ASA40331.1
Gt5GT7	B2NID7.1
NtGT2	BAB88935.1
AmUGT73A9	BAG31950.1
AmUGT88D4	BAG31945.1

Table S4. Result of ^{1}H-NMR and ^{13}C-NMR for the product Q-g-r-g*.

^{1}H-NMR (500 MHz, Methanol-d4)				
Chemical shift (ppm)	Multiplicity	Coupling constant(J, Hz)	Integration	Assignment
7.69	d	2.2	1H	C(2)-H
7.63	dd	8.4, 2.2	1H	C(6)-H
6.88	d	8.4	1H	C(5)-H
6.42	d	2.1	1H	C(8)-H
6.22	d	2.1	1H	C(6)-H
5.09	d	7.7	1H	C(1)-H
4.56	d	1.8	1H	C(1)-H
4.43	d	7.8	1H	C(1)-H
3.94	dd	3.4, 1.7	1H	C(2)-H
3.81	dd	-	1H	C(6A)-H
3.76	dd	-	1H	C(6A)-H
3.73	dd	-	1H	C(6B)-H
3.64	dd	-	1H	C(3)-H
3.52	dd	-	1H	C(5)-H
3.48	m	-	1H	C(6)-H
3.47	m	-	2H	C(2)-H, C(4)-H
3.44	m	-	1H	C(6)-H
3.43	m	-	1H	C(3)-H
3.41	m	-	1H	C(4)-H
3.38	m	-	1H	C(4)-H
3.34	m	-	1H	C(5)-H
3.28	m	-	1H	C(2)-H
3.25	m	-	1H	C(5)-H
1.11	d	6.0	3H	C(6)-H

^{13}C-NMR (126 MHz, Methanol-d4)			
Chemical Shift (ppm)	Assignment	Chemical Shift (ppm)	Assignment
179.39	C-4	99.98	C-6
166.06	C-7	94.97	C-8
162.97	C-5	83.09	C-3
159.38	C-2	78.20	C-5
158.57	C-9	77.58	C-5 , C-3
149.78	C-4	77.19	C-3
145.81	C-3	75.72	C-2
135.70	C-3	75.46	C-2
123.52	C-6	72.59	C-4, C-4
123.10	C-1	71.37	C-2
117.79	C-2	70.85	C-4
116.06	C-5	69.38	C-5
105.62	C-1, C-1	68.81	C-6
104.91	C-10	62.05	C-6
102.35	C-1	17.94	C-6

*. d, dd and m denoted doublet, doublet of doublets and multiplet, respectively. 1H, 2H, and 3H represented one proton, two equivalent protons, and three equivalent protons, respectively.

Table S5. Raw and normalized expression levels of the *CsFGT* in the leaves harvested from different cultivars*.

Sample names	Relative quantity (RQ)	Normalized RQ
FD1	316.98±10.05	1.00±0.03
FD2	298.96±11.29	0.94±0.04
FD3	296.88±15.45	0.94±0.05
FD4	284.72±5.74	0.90±0.02
LJ1	282.31±11.48	0.89±0.04
LJ2	253.56±26.74	0.80±0.08
LJ3	221.39±9.78	0.70±0.03
LJ4	271.00±28.25	0.85±0.09
FX1	209.98±16.95	0.66±0.05
FX2	147.37±51.86	0.46±0.16
FX3	231.34±85.31	0.73±0.27
FX4	142.46±66.15	0.45±0.21
ZJ1	127.75±27.44	0.40±0.09
ZJ2	145.31±16.63	0.46±0.05
ZJ3	202.04±15.14	0.64±0.05
ZJ4	233.52±69.03	0.74±0.22
JX1	146.30±15.85	0.46±0.05
JX2	117.59±5.74	0.37±0.02
JX3	122.21±34.06	0.39±0.11
JX4	139.08±25.90	0.44±0.08
YM1	190.81±20.81	0.60±0.07
YM2	111.86±15.89	0.35±0.05
YM3	162.05±5.75	0.51±0.02
YM4	45.11±3.59	0.14±0.01
JGDB1	3.19±2.64	0.01±0.01
JGDB2	3.61±0.84	0.01±0.00
JGDB3	8.10±4.88	0.03±0.02
JGDB4	14.86±1.89	0.05±0.01
DJBH1	10.72±1.63	0.03±0.01
DJBH2	30.51±8.03	0.10±0.03
DJBH3	23.72±13.06	0.07±0.04
DJBH4	2.16±1.82	0.01±0.01
XGYH1	1.58±0.33	0.00±0.00
XGYH2	2.79±0.14	0.01±0.00
XGYH3	3.18±0.31	0.01±0.00
XGYH4	2.12±0.23	0.01±0.00
XY1	9.22±4.70	0.03±0.01
XY2	11.69±3.41	0.04±0.01
XY3	10.64±1.75	0.03±0.01
XY4	7.86±0.49	0.02±0.00
YK1	5.49±0.35	0.02±0.00
YK2	5.30±0.56	0.02±0.00
YK3	7.54±5.88	0.02±0.02
YK4	5.47±2.13	0.02±0.01
YG1	2.28±1.81	0.01±0.01
YG2	36.46±5.26	0.12±0.02
YG3	10.57±0.51	0.03±0.00
YG4	16.97±0.11	0.05±0.00
ML1	9.96±4.67	0.03±0.01
ML2	8.77±0.95	0.03±0.00
ML3	6.62±2.22	0.02±0.01
ML4	5.74±2.62	0.02±0.01

*. All the shoots (one bud and four leaves) with a similar maturity were collected from Tea Science Research Institute of Pu'er City, Yunnan Province, and 1-4 for each cultivar indicated the leaf position under the apical bud of the shoot.

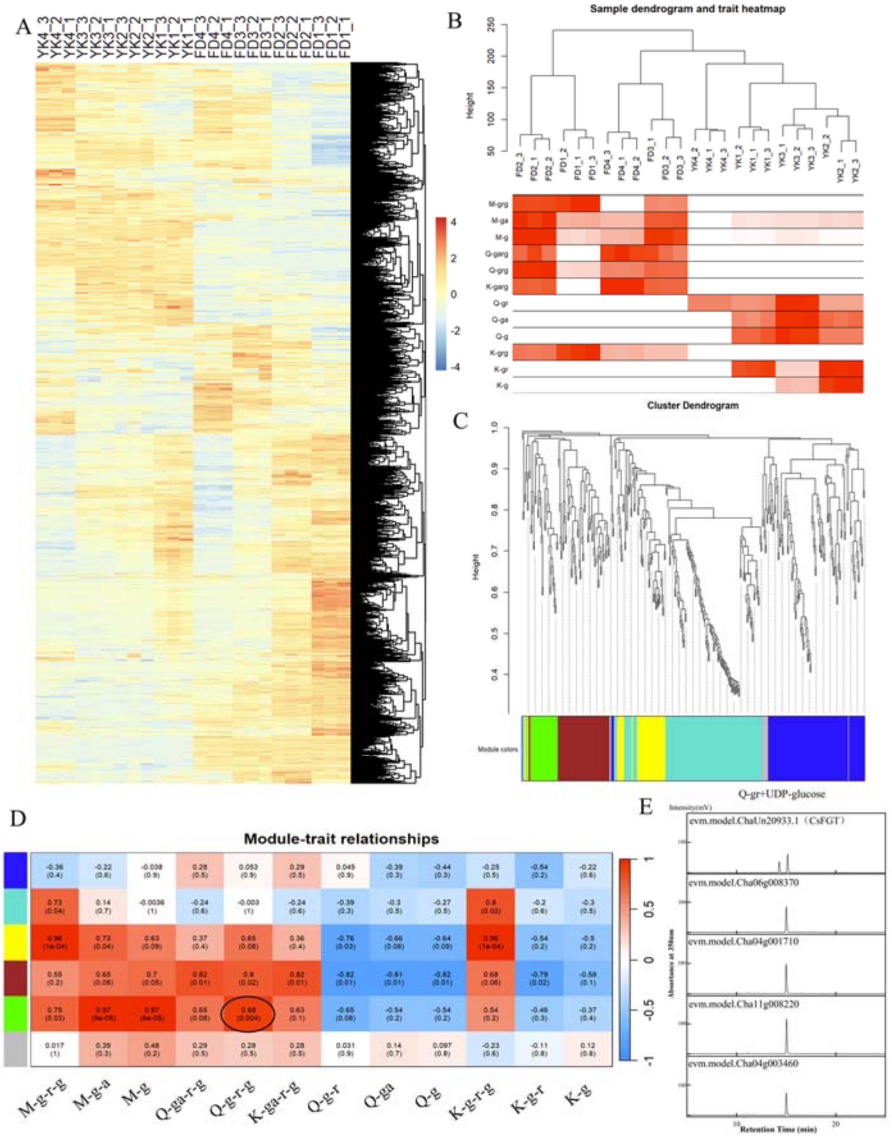

Fig. S1. Screening out the *CsFGT* contributing to Q-g-r-g biosynthesis through WGCNA. A. Cluster heatmap according the expression levels of the DEGs. B. Cluster heatmap according to the content of the metabolites in different tea samples. C. The cutting cluster tree of gene co-expression network. D. Heatmap of correlation coefficient between the expression level of the genes in co-expression network module and the content of the flavonol glycosides. E. The enzyme activity of five candidate *UGTs*. FD1_x~FD4_x and YK1_x~YK4_x indicated the _x repeat of the 1st -4th leaf down the apical bud harvested from cultivar ‘Fudingdabaicha’ (FD) and ‘Yunkang 10#’ (YK). The WGCNA results were plotted using R-Studio analysis, and the trait used for association analysis was the content of flavonol glycosides. The filtering principle of soft threshold was to make the constructed network more consistent with the characteristics of scale-free network. The five candidate genes screened by WGCNA were evm.model.ChaUn20933.1 (CsFGT, OR487152), evm.model.Cha06g008370 (Q66PF3), evm.model.Cha04g001710 (D4Q9Z4), evm.model.Cha11g008220 (O64732) and evm.model.Cha04g003460 (Q9AT45). Five candidate *UGTs* were obtained from the green module, rutin and UDP-glucose were used as sugar acceptor and donor, and only one gene (the *CsFGT*) could catalyze the biosynthesis of Q-g-r-g.

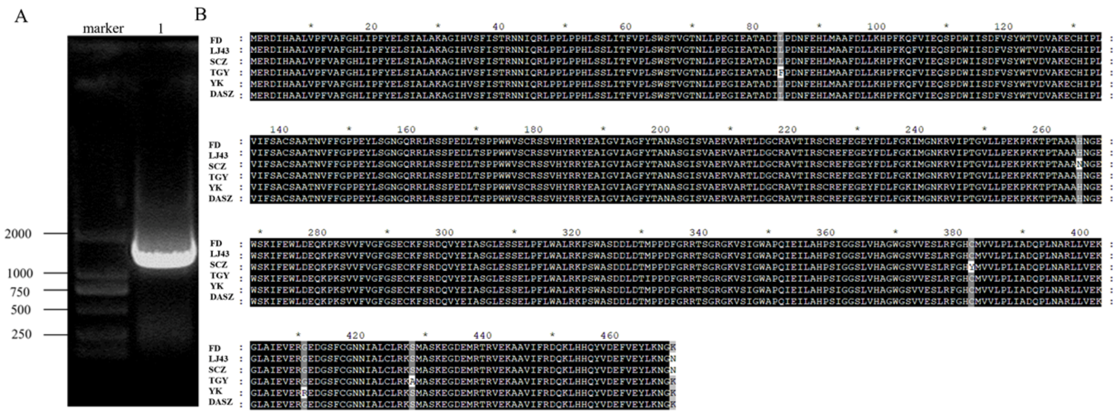

Fig. S2. Gene cloning and comparison of the deduced amino acid sequences among different cultivars. A. RT-PCR product of the *CsFGT* obtained from FD (‘Fudingdabaicha’). M, 2000bp DNA marker; lane 1, *CsFGT*(1410bp). B. Similarity of the deduced amino acid sequences among FD, LJ43 (‘Longjing43’), SCZ (‘Shuchazao’) and TGY (‘Tieguanyin’), YK (‘Yunkang10’) and ‘DASZ’. The cultivars FD, LJ43, SCZ and TGY belonged to *sinensis* variety with small leaf; while YK belonged to *assamica* variety with big leaf; ‘DASZ’ stood for wild tea plant with big leaf. Amino acid sequences of the other cultivars had been obtained through screening of the genomic data, except that of the FD.

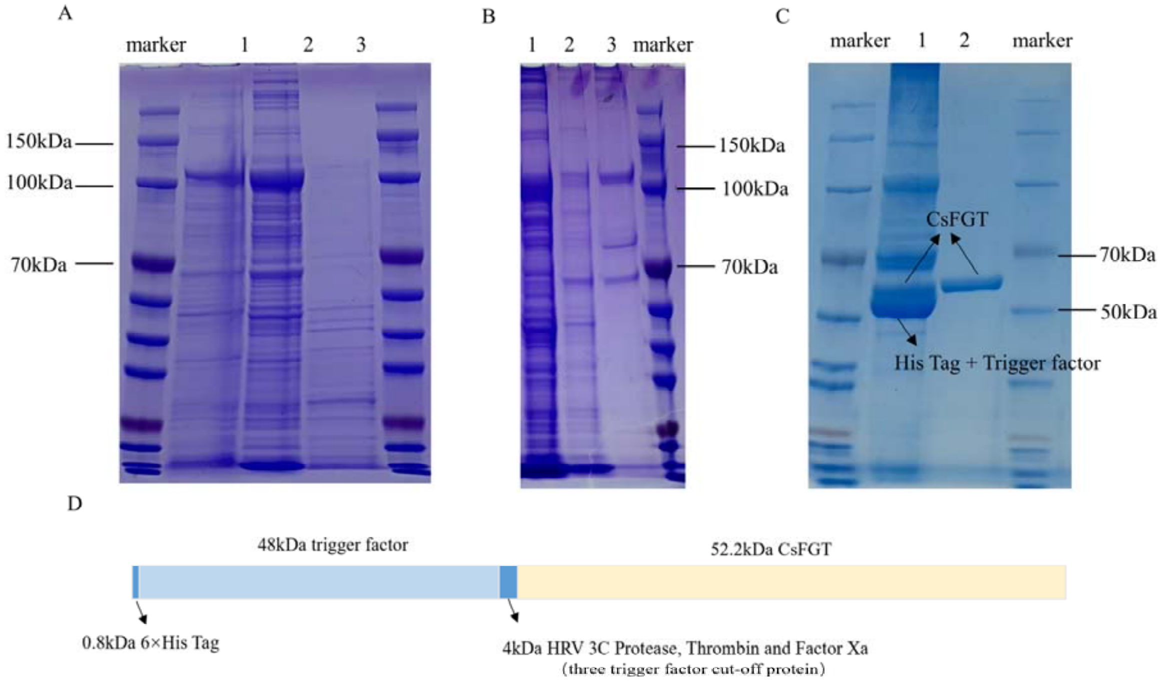

Fig. S3. SDS-PAGE analysis of recombinant CsFGT. The 10 % polyacrylamide gel was used to separate the proteins, and molecular weight of the target recombinant protein was ~105 kDa (52.2 kDa CsFGT fused with 4 kDa trigger factor cut-off polypeptide, 48 kDa trigger factor and 0.8 kDa His·tag). A. Marker, protein molecular weight marker; lane 1, protein in the supernatant obtained from the induced cells; lane 2, total proteins obtained from the induced cells; lane 3, total proteins obtained from the un-induced cells. B. lane 1, supernatant proteins before purification; lane 2, proteins in the effluent during washing stage of purification (wash buffer was used to wash out unbound non-recombinant proteins); lane 3, protein in the eluate (the purified recombinant CsFGT) after elution with elute. C. lane 1, recombinant CsFGT digested by HRV3C overnight; lane 2, CsFGT without His·tag and trigger factor. D. Schematic diagram of recombinant CsFGT protein composition.

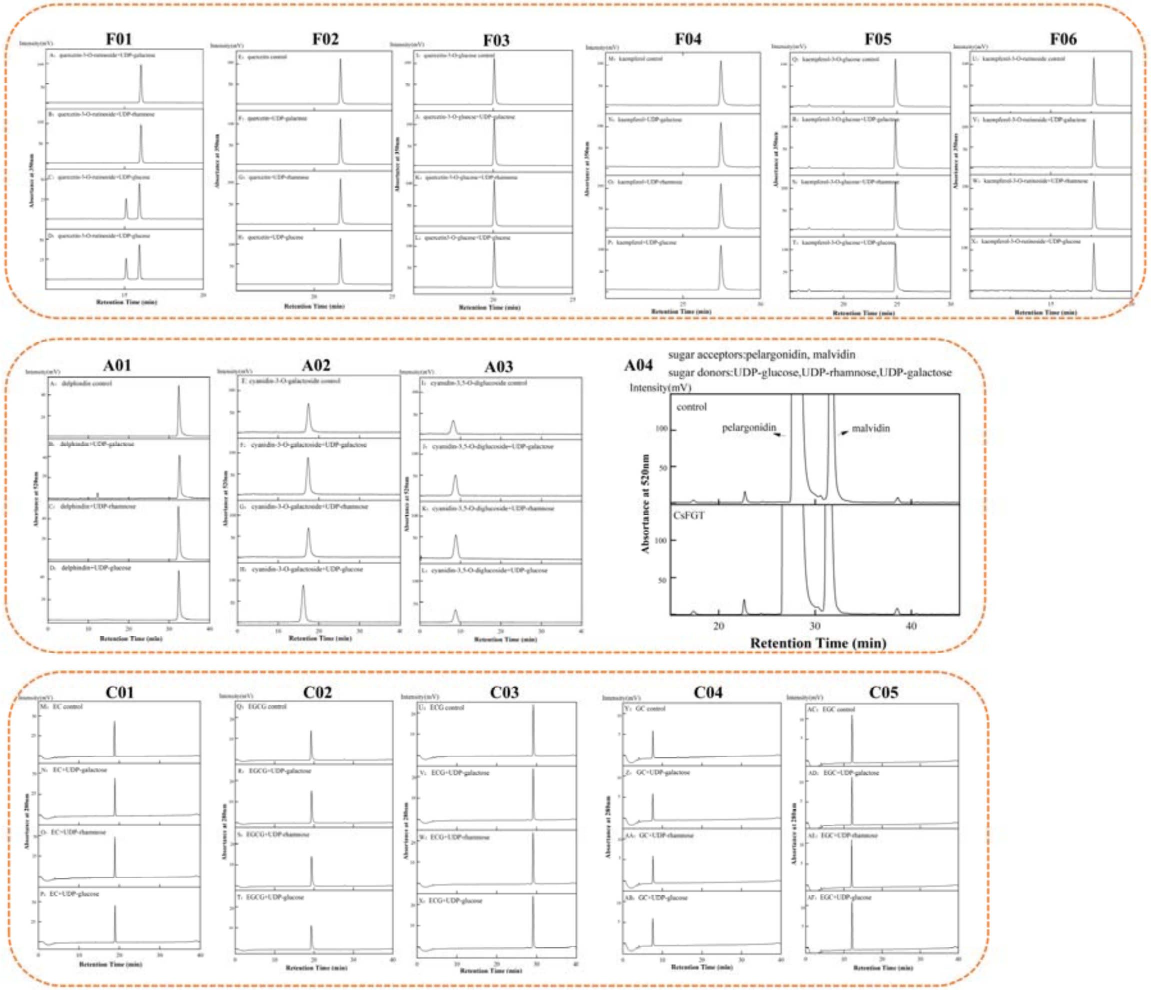

Fig. S4. The glycosyl-transfer activity of the expressed target protein towards different sugar donors and acceptors. The product of the reactions had been monitored by HPLC. F01(A), rCsFGT+quercetin-3-O-rutinoside+UDP-galactose; F01(B), rCsFGT+quercetin-3-O-rutinoside+ UDP-rhamnose; F01(C), rCsFGT+quercetin-3-O-rutinoside+UDP-glucose; F01(D), the purified CsFGT (removal of Hig·tag and trigger factor) +quercetin-3-O-rutinoside+UDP-glucose. F02(E), rCsFGT + quercetin; F02(F), rCsFGT + quercetin+UDP-galactose; F02(G), rCsFGT + quercetin+ UDP-rhamnose; F02(H), rCsFGT + quercetin+ UDP-glucose. F03(I), rCsFGT + quercetin-3-O-glucose; F03(J), rCsFGT + quercetin-3-O-glucose +UDP-galactose; F03(K), rCsFGT + quercetin-3-O-glucose + UDP-rhamnose; F03(L), rCsFGT + quercetin-3-O-glucose + UDP-glucose. F04(M), rCsFGT + kaempferol; F04(N), rCsFGT + kaempferol +UDP-galactose; F04(O), rCsFGT + kaempferol + UDP-rhamnose; F04(P), rCsFGT + kaempferol + UDP-glucose. F05(Q), rCsFGT + kaempferol-3-O-glucose; F05(R), rCsFGT + kaempferol-3-O-glucose +UDP-

galactose; F05(S), rCsFGT + kaempferol-3-O-glucose + UDP-rhamnose; F05(T), rCsFGT + kaempferol-3-O-glucose + UDP-glucose. F06(U), rCsFGT + kaempferol-3-O-rutinoside; F06(V), rCsFGT + kaempferol-3-O-rutinoside +UDP-galactose; F06(W), rCsFGT + kaempferol-3-O-rutinoside + UDP-rhamnose; F06(X), rCsFGT + kaempferol-3-O-rutinoside + UDP-glucose. A01(A), rCsFGT + delphindin; A01(B), rCsFGT + delphindin +UDP-galactose; A01(C), rCsFGT + delphindin + UDP-rhamnose; A01(D), rCsFGT + delphindin + UDP-glucose. A02(E), rCsFGT + cyanidin-3-O-galactoside; A02(F), rCsFGT + cyanidin-3-O-galactoside +UDP-galactose; A02(G), rCsFGT + cyanidin-3-O-galactoside + UDP-rhamnose; A02(H), rCsFGT + cyanidin-3-O-galactoside + UDP-glucose. A03(I), rCsFGT + cyanidin-3,5 -O-diglucoside; A03(J), rCsFGT + cyanidin-3,5 -O-diglucoside +UDP-galactose; A03(K), rCsFGT + cyanidin-3,5 -O-diglucoside + UDP-rhamnose; A03(L), rCsFGT + cyanidin-3,5 -O-diglucoside + UDP-glucose. A04(control), pelargonidin /malvidin +UDP-galactose/ UDP-rhamnose/ UDP-glucose; A04(rCsFGT), rCsFGT+ pelargonidin /malvidin +UDP-galactose/ UDP-rhamnose/ UDP-glucose. C01(A), rCsFGT + epicatechin(EC); C01(B), rCsFGT + EC +UDP-galactose; C01(C), rCsFGT + EC + UDP-rhamnose; C01(D), rCsFGT + EC + UDP-glucose. C02(E), rCsFGT + epigallocatechin gallate (EGCG); C02(F), rCsFGT + EGCG +UDP-galactose; C02(G), rCsFGT +EGCG + UDP-rhamnose; C02(H), rCsFGT + EGCG + UDP-glucose. C03(I), rCsFGT + epicatechin gallate (ECG); C03(J), rCsFGT + ECG +UDP-galactose; C03(K), rCsFGT + ECG + UDP-rhamnose; C03(L), rCsFGT + ECG + UDP-glucose. C04(M), rCsFGT + gallocatechin (GC); C04(N), rCsFGT + GC +UDP-galactose; C04(O), rCsFGT + GC + UDP-rhamnose; C04(P), rCsFGT + GC + UDP-glucose. C05(Q), rCsFGT + epigallocatechin (EGC); C05(R), rCsFGT + EGC +UDP-galactose; C05(S), rCsFGT + EGC + UDP-rhamnose; C05(T), rCsFGT + EGC+ UDP-glucose.

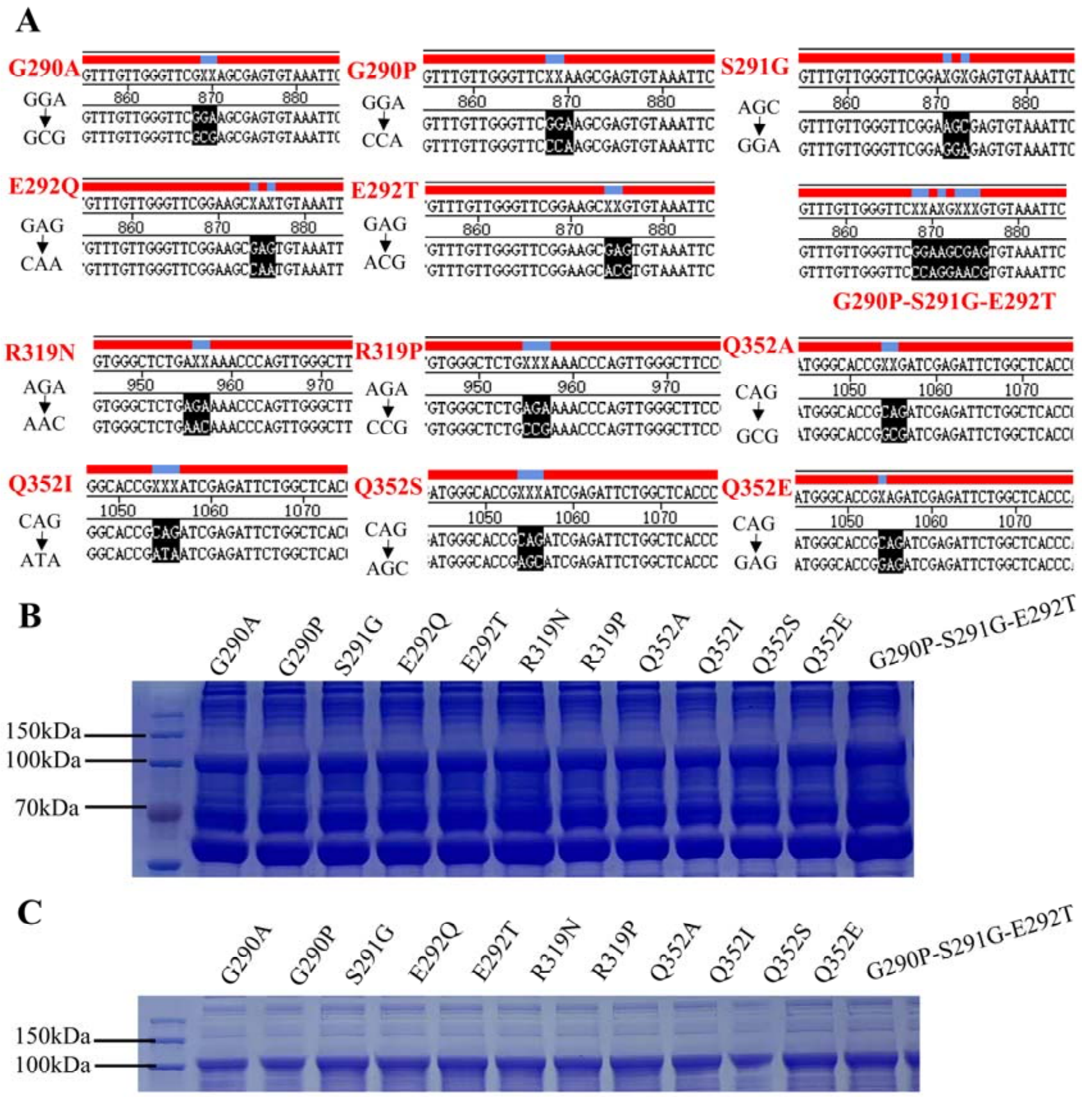

Fig. S5. Codon mutation confirmation and SDS-PAGE analysis of the mutant proteins. A. site-directed mutagenesis confirmation through sequencing. B. Unpurified soluble proteins extracted from DE3 cells harboring different CsFGT mutants. The molecular weight of the target protein was ~105kDa (52.2kDa mutant protein fused with 4kDa trigger factor cut-off polypeptide, 48 kDa trigger factor and 0.8kDa His·tag). C. The mutated proteins after purification.

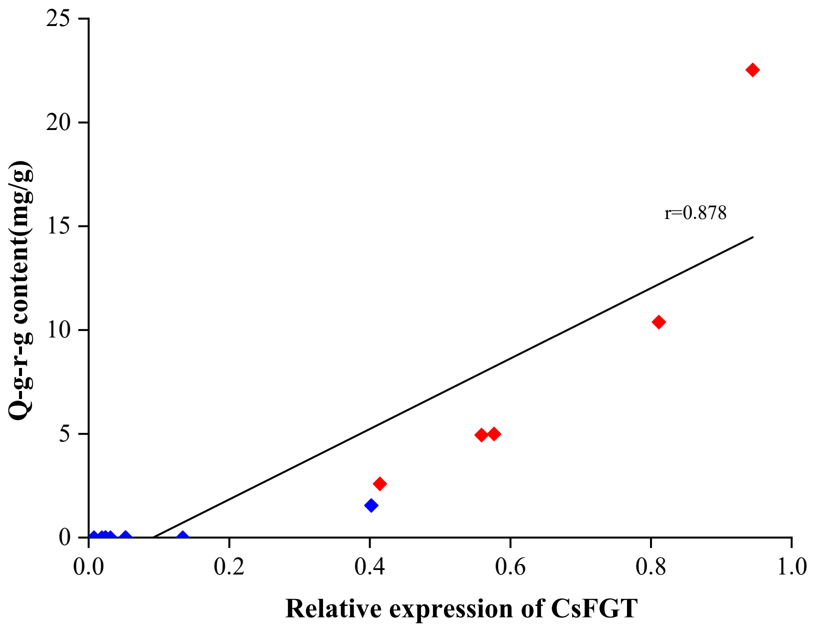

Fig. S6. Relationship between *CsFGT* expression and Q-g-r-g content in the leaves of different tea cultivars. Red diamond indicated the cultivar belonging to *sinensis* variety and blue diamond indicated the cultivar belonging to *assamica* variety.

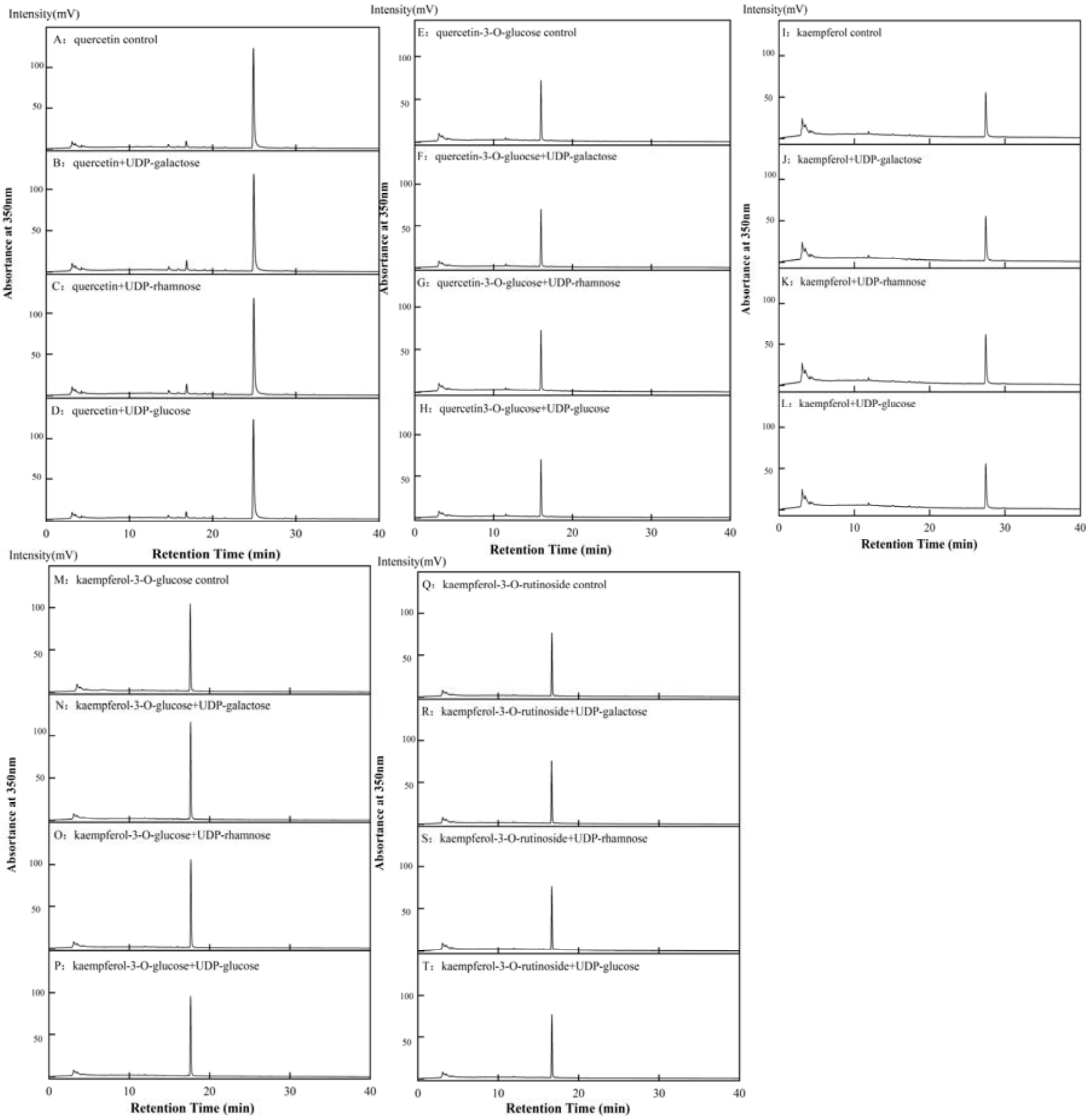

Fig. S7. The activity of G290P-S291G-E292T mutant towards different sugar acceptors and donors. A. quercetin was used as sugar acceptor and none of sugar donor was used; B. quercetin and UDP-galactose were used as sugar acceptor and donor; C. quercetin and UDP-rhamnose were used as sugar acceptor and donor; D. quercetin and UDP-glucose were used as sugar acceptor and donor; E. quercetin-3-O-glucose was used as sugar acceptor and none of sugar donor was used; F. quercetin-3-O-glucose and UDP-galactose were used as sugar acceptor and donor; G. quercetin-3-O-glucose and UDP-rhamnose were used as sugar acceptor and donor; H. quercetin-3-O-glucose and UDP-glucose were used as sugar acceptor and donor; I. kaempferol was used as sugar acceptor and none of sugar donor was used; J. kaempferol and UDP-galactose were used as sugar acceptor and donor; K. kaempferol and UDP-rhamnose were used as sugar acceptor and donor; L. kaempferol and UDP-glucose were used as sugar acceptor and donor; M. kaempferol-3-O-glucose was used as sugar acceptor and none of sugar donor was used; N. kaempferol-3-O-glucose and UDP-galactose were used as sugar acceptor and donor; O. kaempferol-3-O-glucose and UDP-rhamnose were used as sugar acceptor and donor; P. kaempferol-3-O-glucose and UDP-glucose were used as sugar acceptor and donor; Q. kaempferol-3-O-rutinoside was used as sugar acceptor and none of sugar donor was used; R. kaempferol-3-O-rutinoside and UDP-galactose were used as sugar acceptor and donor; S. kaempferol-3-O-rutinoside and UDP-rhamnose were used as sugar acceptor and donor; T. kaempferol-3-O-rutinoside and UDP-glucose were used as sugar acceptor and donor.

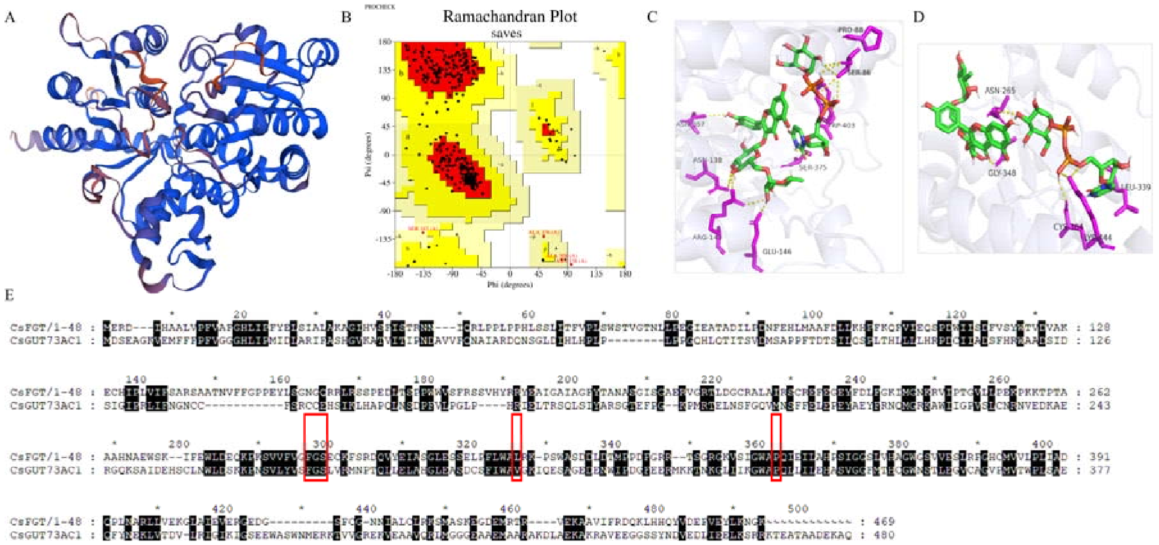

Fig. S8. Explanation for the difference in catalysis property of CsUGT73AC15 in comparison with CsFGT. A. Homologous modeling of CsUGT73AC15. B. Ramachandran plot of the CsUGT73AC15. C. Molecular docking of the CsUGT73AC15 with the rutin and UDP-glucose. D. Molecular docking of the CsUGT73AC15 with the kaempferol 3-*O*-rutinoside and UDP-glucose. E. Sequence alignment of CsUGT73AC15 and CsFGT. The red boxes showed the key amino acid residues identified in CsFGT.
